# Rational affinity maturation of anti-amyloid antibodies with high conformational and sequence specificity

**DOI:** 10.1016/j.jbc.2021.100508

**Published:** 2021-03-04

**Authors:** Alec A. Desai, Matthew D. Smith, Yulei Zhang, Emily K. Makowski, Julia E. Gerson, Edward Ionescu, Charles G. Starr, Jennifer M. Zupancic, Shannon J. Moore, Alexandra B. Sutter, Magdalena I. Ivanova, Geoffrey G. Murphy, Henry L. Paulson, Peter M. Tessier

**Affiliations:** 1 Department of Chemical Engineering, University of Michigan, Ann Arbor, Michigan, USA; 2 Biointerfaces Institute, University of Michigan, Ann Arbor, Michigan, USA; 3 Department of Pharmaceutical Sciences, University of Michigan, Ann Arbor, Michigan, USA; 4 Department of Neurology, University of Michigan, Ann Arbor, Michigan, USA; 5 Protein Folding Disease Initiative, University of Michigan, Ann Arbor, Michigan, USA; 6 Department of Molecular & Integrative Physiology, University of Michigan, Ann Arbor, Michigan, USA; 7 Biophysics Program, University of Michigan, Ann Arbor, Michigan, USA; 8 Michigan Alzheimer’s Disease Center, University of Michigan, Ann Arbor, Michigan, USA; 9 Department of Biomedical Engineering, University of Michigan, Ann Arbor, Michigan, USA

**Keywords:** fibril, aggregate, amyloid, Alzheimer, directed evolution, mAb, scFv, yeast surface display, antibody engineering, neurodegeneration, Aβ, amyloid beta, BSA, bovine serum albumin, CDR, complementarity-determining region, ELISA, enzyme-linked immunosorbent assay, Fc, fragment crystallizable, scFv, single-chain variable fragment, V_H_, variable domain of heavy chain, V_L_, variable domain of light chain

## Abstract

The aggregation of amyloidogenic polypeptides is strongly linked to several neurodegenerative disorders, including Alzheimer’s and Parkinson’s diseases. Conformational antibodies that selectively recognize protein aggregates are leading therapeutic agents for selectively neutralizing toxic aggregates, diagnostic and imaging agents for detecting disease, and biomedical reagents for elucidating disease mechanisms. Despite their importance, it is challenging to generate high-quality conformational antibodies in a systematic and site-specific manner due to the properties of protein aggregates (hydrophobic, multivalent, and heterogeneous) and limitations of immunization (uncontrolled antigen presentation and immunodominant epitopes). Toward addressing these challenges, we have developed a systematic directed evolution procedure for affinity maturing antibodies against Alzheimer’s Aβ fibrils and selecting variants with strict conformational and sequence specificity. We first designed a library based on a lead conformational antibody by sampling combinations of amino acids in the antigen-binding site predicted to mediate high antibody specificity. Next, we displayed this library on the surface of yeast, sorted it against Aβ42 aggregates, and identified promising clones using deep sequencing. The resulting antibodies displayed similar or higher affinities than clinical-stage Aβ antibodies (aducanumab and crenezumab). Moreover, the affinity-matured antibodies retained high conformational specificity for Aβ aggregates, as observed for aducanumab and unlike crenezumab. Notably, the affinity-maturated antibodies displayed extremely low levels of nonspecific interactions, as observed for crenezumab and unlike aducanumab. We expect that our systematic methods for generating antibodies with unique combinations of desirable properties will improve the generation of high-quality conformational antibodies specific for diverse types of aggregated conformers.

Of the many human disorders facing our society today, neurodegenerative diseases such as Alzheimer’s and Parkinson’s diseases are arguably the most menacing and least treatable (1, 2). These diseases – which are linked to the formation of toxic prefibrillar oligomers and amyloid fibrils – are particularly concerning because their frequency of occurrence is linked to age and, thus, the number of cases is expected to increase as life expectancy increases in the coming years due to significant advances in treating other human disorders such as cancer and heart diseases.

Conformational antibodies specific for different conformers of amyloid-forming proteins are important for detecting, disrupting, and reversing toxic protein aggregation (3, 4). Several previous reports have demonstrated creative methods for using immunization (4, 5, 6, 7, 8, 9, 10, 11, 12), autoantibody screening (5, 13, 14, 15, 16, 17, 18, 19, 20, 21, 22), directed evolution (23, 24, 25, 26), and rational design methods (27, 28, 29, 30, 31, 32) for generating such antibodies. Despite this progress, there are several common problems associated with generating conformational antibodies against amyloid-forming proteins. First, the nature of amyloidogenic antigens is extremely complex and particularly unattractive for typical antibody selection methods due to their insolubility, heterogeneity in terms of size and conformation, hydrophobicity, and multivalency. Second, the use of immunization to generate such antibodies is limited due to uncontrolled presentation of aggregated antigens to the immune system and immunodominant epitopes. Third, the use of conventional directed evolution methods such as yeast surface display is limited by the inability to use fluorescence-activated cell sorting due to the lack of soluble antigens.

These and many other challenges typically result in antibodies that recognize protein aggregates with either conformational specificity [*e.g.*, common fibril or oligomer structure (5, 6, 20)] or sequence specificity (*e.g.*, linear peptide epitopes) but not both. Even in cases where antibodies with strict conformational and sequence specificity have been identified [*e.g.*, (10, 16, 22, 33, 34)], these approaches typically require extensive secondary screening to identify such rare variants and are not readily extendable to generate conformational antibodies against different sites in the same protein or other proteins in a systematic, efficient, and predictable manner.

Toward the goal of rational and efficient methods for generating high-quality antibodies with strict conformational and sequence specificity, we have previously developed directed evolution methods for discovering lead antibodies with high conformational specificity (23). Our approach involves designing single-chain (scFv) antibody libraries with focused mutagenesis in the most important antibody complementarity-determining region (CDR) that typically governs antigen binding (heavy chain CDR3). We sampled combinations of mutations that are most commonly observed in natural antibodies based on tens of thousands of human antibody CDRs (35). From such libraries, we identified an attractive lead antibody (AF1) that recognizes amyloid fibrils of the Aβ42 peptide with high conformational and sequence specificity (23). This antibody displays much weaker affinity for disaggregated Aβ42 and extremely low levels of nonspecific binding even at high antibody concentrations (100 nM). Interestingly, the low levels of nonspecific binding for AF1 is similar to that of several highly specific, clinical-stage antibodies (36).

Nevertheless, the apparent affinity of AF1 for Aβ42 fibrils is modest (EC_50_ of ∼100 nM) and at least an order of magnitude weaker than other clinical-stage antibodies that target Aβ42 aggregates. Therefore, we sought to affinity mature AF1 against Aβ fibrils to increase affinity while maintaining strict conformational and sequence specificity as well as low levels of nonspecific binding.

To accomplish this, there are several challenges that must be addressed. The first and most significant challenge is that most mutations that increase the affinity of such conformational antibodies also increase specific interactions with soluble Aβ (reduced conformational specificity) or nonspecific interactions (reduced sequence specificity) or both. A second key challenge is that the multivalent nature of protein aggregates frustrates the selection of affinity-enhancing mutations due to avidity effects. A third challenge is the selection of antibody sites to mutate as well as sets of mutations to sample in order to maximize the likelihood of obtaining matured antibody variants with high specificity and low levels of nonspecific interactions. Here we report an integrated approach for affinity maturing conformational antibodies specific for Aβ fibrils and demonstrate that this approach results in antibody variants with favorable combinations of binding properties relative to Aβ clinical-stage antibodies.

## Results

### Antibody library design and identification of affinity-matured candidates

**Figure 1:**
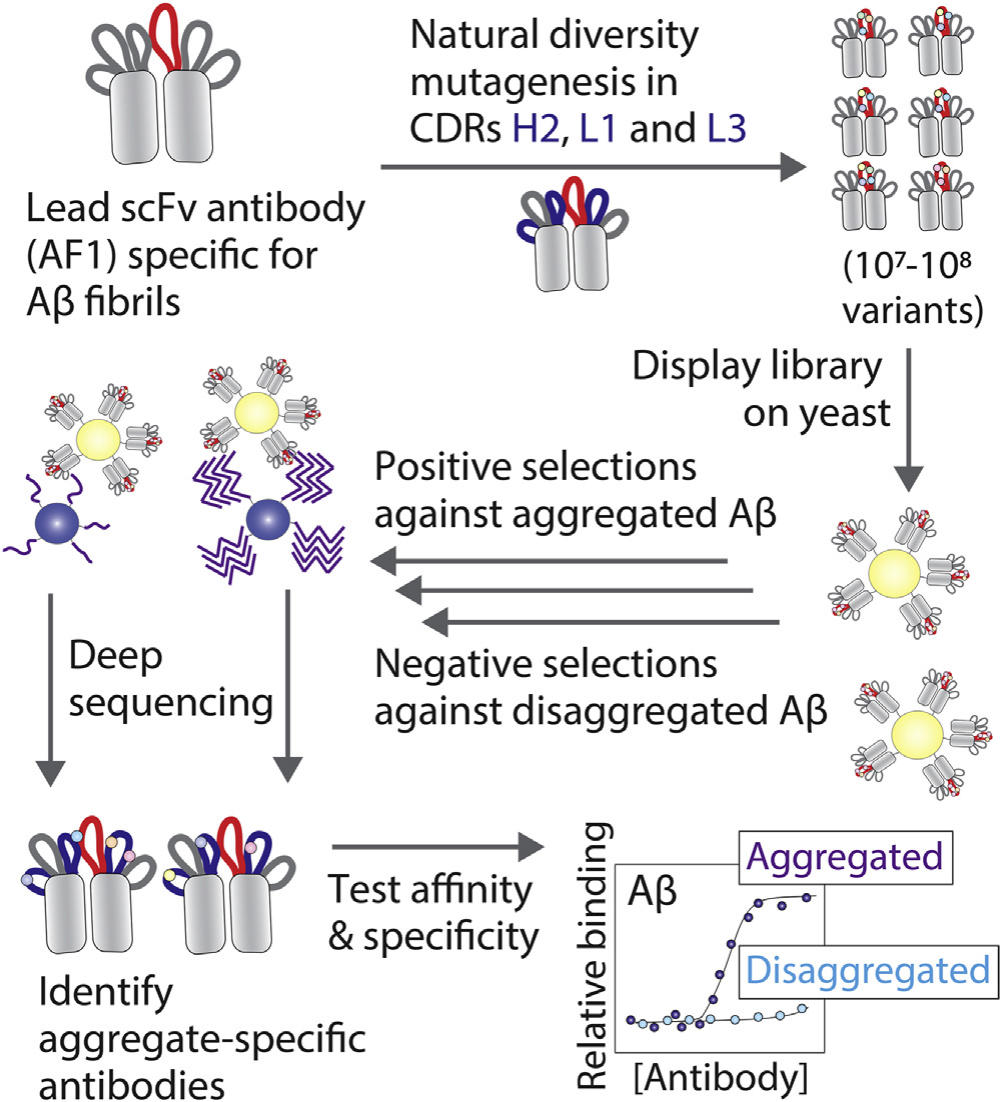
**Proposed method for systematically maturing the affinity and specificity of Aβ amyloid antibodies.** A lead single-chain antibody fragment (scFv) specific for Aβ fibrils was mutated by targeting solvent-exposed and naturally diverse sites in three complementarity-determining regions (CDRs), including heavy-chain CDR2 (H2) and light-chain CDRs 1 (L1) and 3 (L3). The library was displayed on yeast and sorted negatively against disaggregated Aβ and positively against aggregated Aβ using magnetic-activated cell sorting. The enriched libraries were subjected to deep sequencing, and clones with mutations predicted to be favorable were evaluated in terms of their affinities and conformational specificities.

Our strategy for systematic affinity maturation of a lead Aβ conformational antibody is summarized in Figure 1. The first step in this process is to design an antibody library that preserves the antigen recognition activity of the lead antibody (AF1) while identifying sites in the CDRs for affinity maturation. Given that AF1 was generated by directed mutagenesis in heavy chain CDR3, we sought to identify sites in the other five CDRs for further mutagenesis. However, there are a large number of potential CDR sites to mutate (54 positions in the five CDRs) and a daunting number of theoretically possible antibody variants (>10^70^ variants).

To limit the library design to a size that can be evaluated using standard display methods such as yeast surface display (∼10^7^–10^9^ variants), we sought to identify the most attractive subset of CDR sites and subset of residues per site that met a number of design criteria. First, we reasoned that the most naturally diverse sites in human antibodies are the most attractive ones for mutagenesis because they are most likely to be solvent exposed and positioned for productive engagement of the antigen while being least likely to adversely impact protein stability (35). We only considered CDR sites in which the most common residue on average in human antibodies, as judged by the AbYsis database of tens of thousands of human antibodies (37), was present at a frequency of <50%.

Next, we prioritized the remaining CDR sites for mutagenesis with the goal of sampling combinations of 4 to 6 residues per site that included the wild-type residue and combinations of residues expected to lead to high antibody specificity in addition to high affinity. The lead AF1 clone possessed five Asp and five Tyr residues in heavy chain CDR3, and we previously found that removal of either type of residue from this CDR reduced specificity and increased nonspecific binding (38). Therefore, we identified sites in the other CDRs that were compatible with encoding the wild-type residue and at least one of these residues (Asp or Tyr) as well as other residues that are most common in human antibodies using degenerate codons (39). Third, we eliminated degenerate codons that included positively charged residues (Arg, Lys, and His) because we and others have shown that excessive positive charge in the antigen-binding site is linked to increased risk for nonspecific interactions (40, 41, 42, 43, 44, 45, 46). We also eliminated degenerate codons that encoded stop codons and minimized the number of Cys-encoding codons while not completely eliminating them. The reason for not completely excluding Cys from the library design is because it is encoded by degenerate codons that include combinations of common CDR residues in human antibodies such as Gly, Tyr, and Asp. Fourth, we selected degenerate codons that maximized the sum of the average frequencies of each residue in human antibodies to maximize coverage of the natural amino acid diversity of human antibodies.

**Figure 2:**
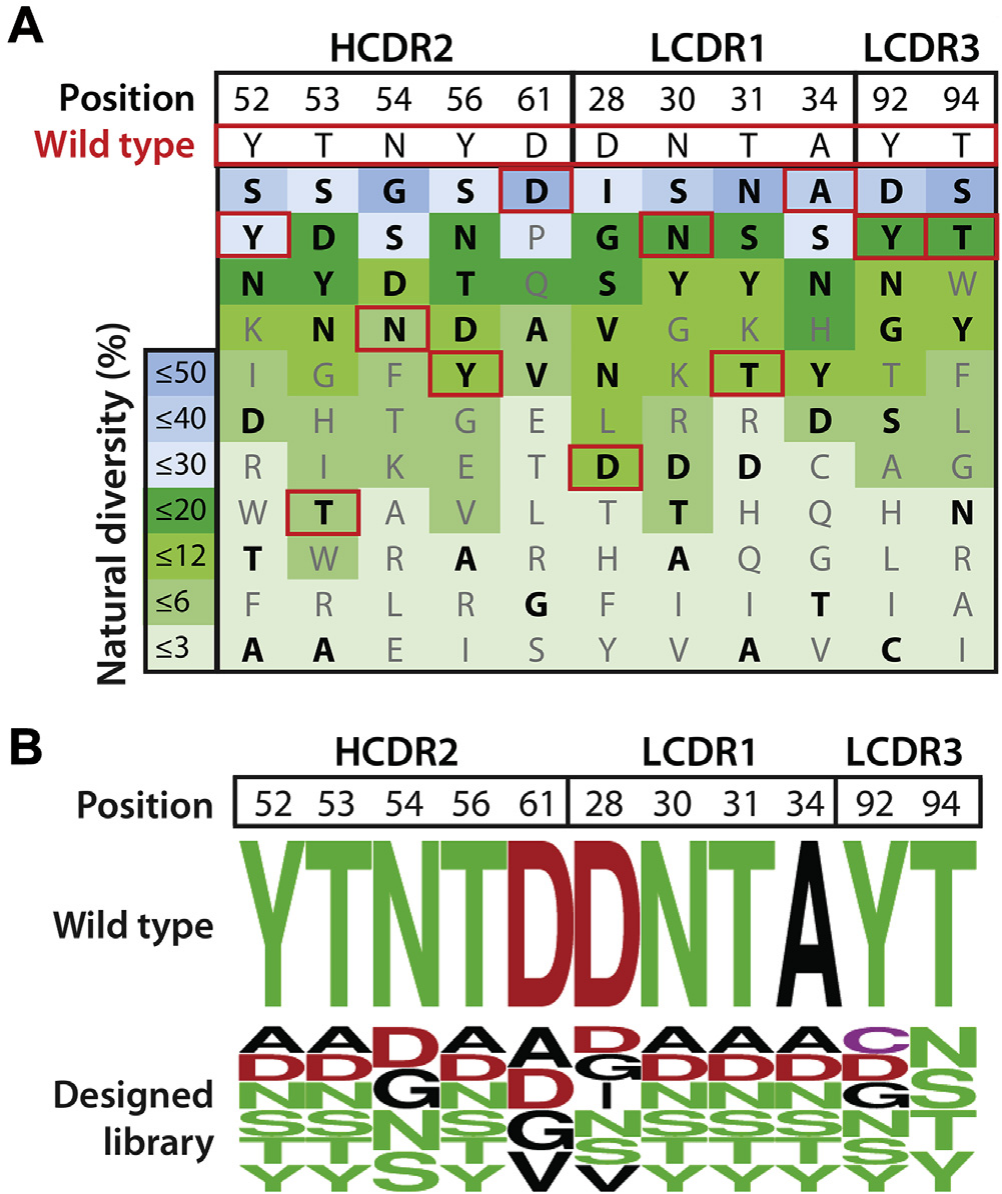
**Design of AF1 antibody sublibrary for affinity maturation that targets naturally diverse and solvent-exposed CDR sites with mutations that are common in human antibodies.***A*, sites in heavy (H2) and light (L1 and L3) chain CDRs for mutagenesis were identified based on their solvent exposure, diversity in human antibodies, and compatibility with sets of mutations most commonly observed in human antibodies. The wild-type residues at each site (boxed in *red*) were included in the library, and the average frequency (%) of each residue observed at each site in human antibodies is color coded. Some of the most common residues in human antibodies were not sampled because they are incompatible with degenerate codons encoding the wild-type residue and other favorable residues. Residues in *black* and *bold text* were sampled at each site. *B*, summary of the designed antibody library at 11 CDR sites that includes the wild-type residue and 3 to 5 mutations that aim to sample combinations of residues most commonly observed in human antibodies. The color codes are *green* for polar residues, *red* for negatively charged residues, *black* for hydrophobic residues, and *purple* for cysteine residues.

Our library design is shown in Figure 2. We identified 11 sites for mutagenesis in three CDRs, namely five sites in heavy chain CDR2, four sites in light chain CDR 1, and two sites in light chain CDR3. At each site, the wild-type residue is boxed in red and the 3 to 5 mutations included in our designs are highlighted as bolded black font (Fig. 2*A*). At each site, the residues are listed in order of most common on average in human antibodies (top) to least common (bottom). For example, at position 52 in heavy chain CDR2, we sampled the wild-type residue (Tyr) along with five other residues that included Asp, two residues common in human antibodies at this position (Ser and Asn), and two residues that are less common but required because of the constraints of degenerate codons. Using a similar strategy at the other ten CDR sites (Fig. 2*B*), the resulting designed library contained 1.1 × 10^8^ theoretical variants.

**Figure 3:**
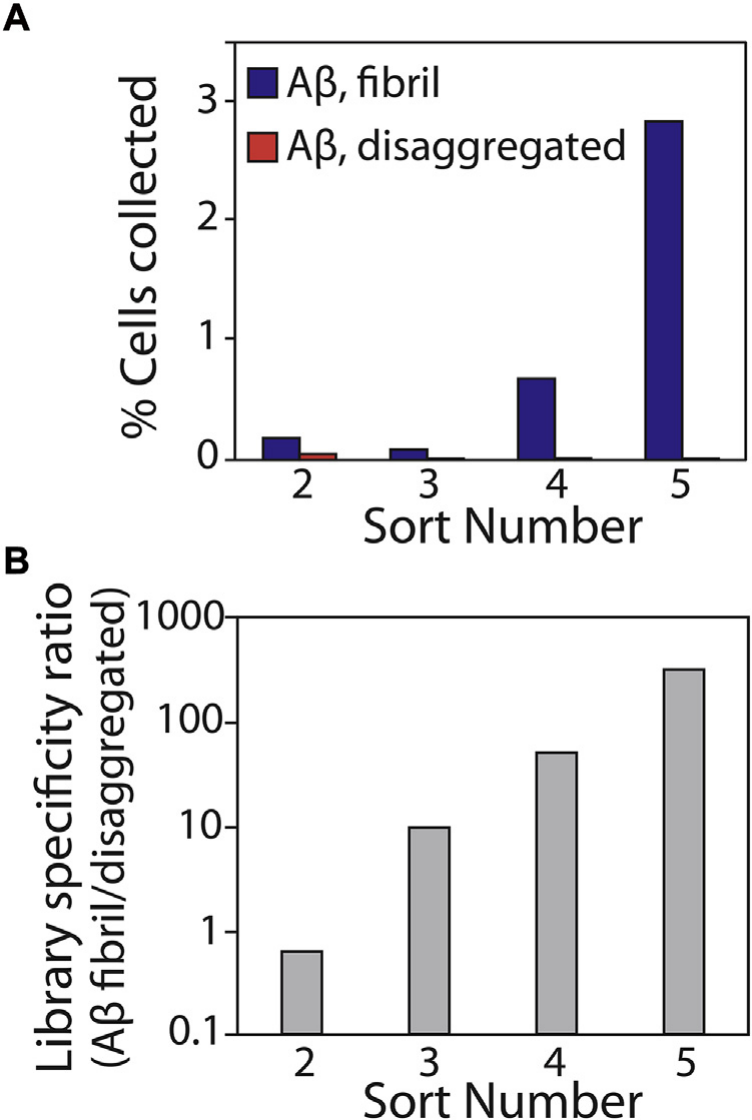
**Summary of the results for sorting the yeast-displayed antibody library against Aβ fibrils.***A*, the library was subjected to five rounds of selection against Aβ fibrils, and the percentage of retained cells was evaluated for both fibrillar and disaggregated Aβ. *B*, the ratio of antibody-displaying yeast cells bound to fibrillar Aβ relative to disaggregated Aβ in each round of selection. In (*A*) and (*B*), 10^7^ antibody-displaying yeast cells were used in rounds 2 to 5 of selection relative to 10^9^ yeast cells in round 1.

Next, we generated the antibody library, displayed it on the surface of yeast as C-terminal Aga2 fusion proteins, and sorted the library against Aβ42 fibrils immobilized on magnetic beads (Fig. 3). To maximize antibody specificity, we performed three negative selections per round of sorting to remove nonspecific antibodies before performing positive selections against Aβ fibrils. In rounds 1 and 2, we performed negative selections against disaggregated (immobilized) Aβ to maximize conformational specificity. In rounds 3 to 5, we performed negative selections against islet amyloid polypeptide (IAPP) fibrils to maximize sequence specificity. After five rounds of sorting, we observed strong enrichment in terms of the percentage of yeast cells that bound to fibrillar Aβ relative to control selections performed against disaggregated Aβ (Fig. 3*A*). The ratio of the number of yeast cells retained against fibrillar Aβ relative to that for disaggregated Aβ was >100 after five rounds of selection (Fig. 3*B*).

**Figure 4:**
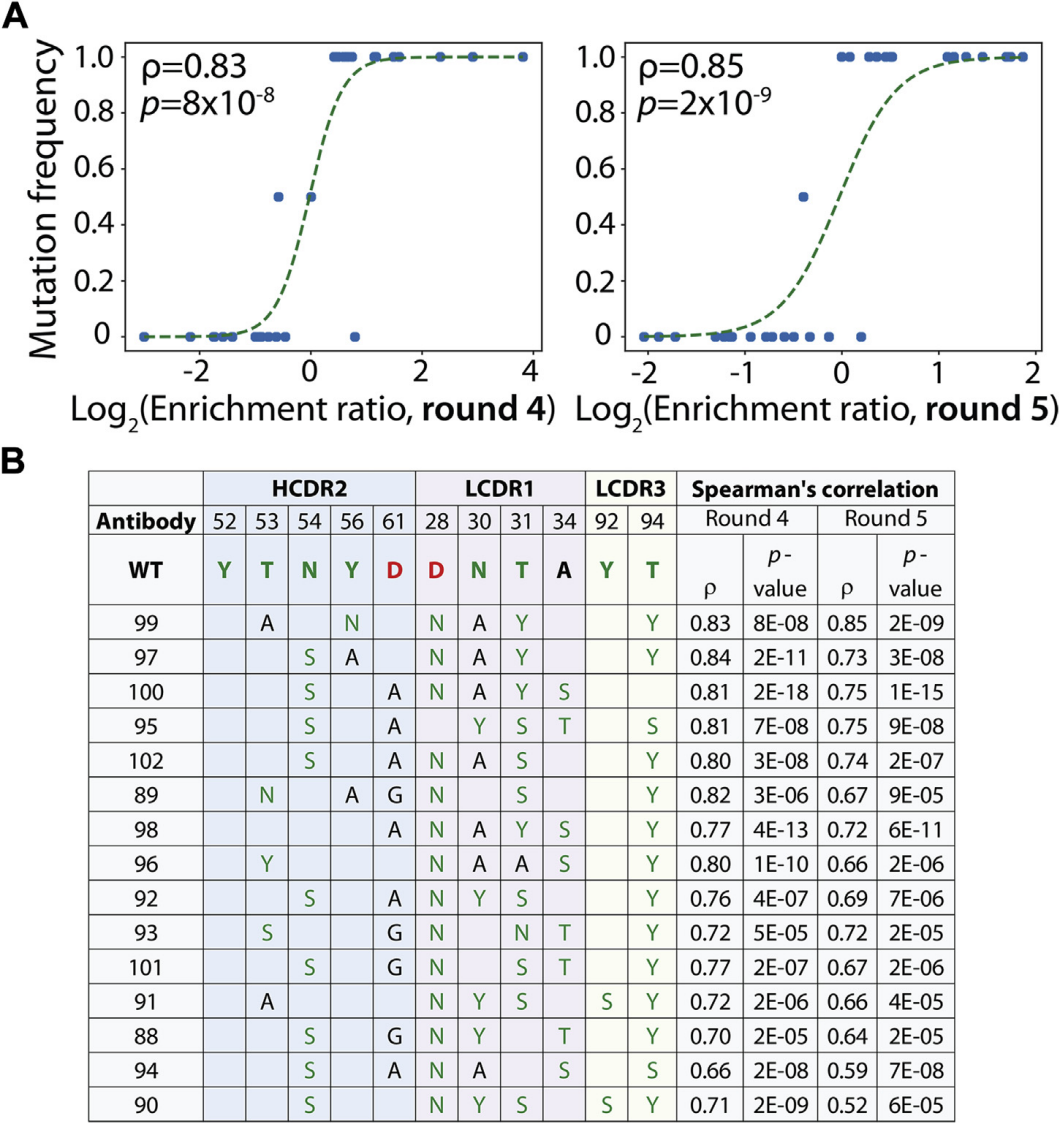
**Identification of sets of affinity-enhancing mutations using deep sequencing.** Antibody libraries were sequenced before and after sorts 4 and 5 against Aβ fibrils, and sets of six mutations were identified that were strongly correlated with increased enrichment relative to wild-type. *A*, correlation between the frequency of clones with a particular set of six mutations (T53A and Y56N in HCDR2, D28N, N30A and T31Y in LCDR1, and T94Y in LCDR3) and enrichment ratios for clones observed with the corresponding mutations. *B*, antibody variants with sets of six mutations that display strong correlation with improved enrichment for recognizing Aβ fibrils relative to wild-type (AF1). In (*A*), the *lines* (logistic regression curves) are guides to the eye. In (*A*) and (*B*), the Spearman correlation coefficients were evaluated using antibody variants with wild-type or mutant residues at the specified positions regardless of their residues at the other five mutated positions. There are 2 to 4 additional mutations not shown for each antibody variant because they are not one of the six mutations most correlated with improved enrichment ratios. Enrichment ratios were calculated as the ratios of the frequencies of each variant observed in the sequencing results for the fibril selections (output) divided by the corresponding values for the input frequencies. The color codes for the amino acids are described in Figure 2.

These promising sorting results led us to sequence the sorted antibody libraries before and after rounds 4 and 5 of selection to better understand mutations most strongly correlated with improved antibody binding (Fig. 4). We identified 7464 unique antibodies using deep sequencing and evaluated correlations between individual mutations or sets of mutations and enrichment ratios for antibody variants with such mutations. Therefore, we evaluated the Spearman correlation coefficients for all possible single and multiple sets of mutations by comparing the enrichment ratios for all antibody variants with either wild-type or mutant residues at these sites regardless of the residues at the other sites. While significant sets of mutations were identified when considering as few as one mutation and as many as nine mutations (the maximum we evaluated), we found that sets of five and six mutations led to the best combination of relatively large numbers of mutant (>10) and wild-type (>10) antibodies per set of mutations, high Spearman correlation values (ρ > 0.5), and high statistical significance (*p*-value < 0.001). Moreover, we found that Spearman correlation coefficients were well correlated between rounds 4 and 5 of sorting, which demonstrates that the deep sequencing results are consistent between multiple rounds of sorting (Fig. S1).

For example, we evaluated a set of six mutations (T53A and Y56N in HCDR2, D28N, N30A and T31Y in LCDR1, and T94Y in LCDR3) by identifying all antibody variants that had these mutations (16 variants) or wild-type residues (16 variants) at these positions regardless of their residues at the other five mutated sites (Fig. 4*A*). We found that this set of mutations resulted in large, positive, and highly significant Spearman correlation coefficients in both rounds 4 (ρ = 0.83 and *p*-value of 8 × 10^−8^) and 5 (ρ = 0.85 and *p*-value of 2 × 10^−9^). We expected that antibody variants with these mutations would display improved antibody affinity.

Several other sets of six mutations were observed that also displayed favorable Spearman correlations, and we selected antibody variants with these mutations for further analysis (Fig. 4*B*). We also identified sets of five mutations with favorable Spearman correlation coefficients that correspond to these same antibody variants (Fig. S2). The antibodies in Figure 4*B* and Fig. S2 had a total of 8 to 10 mutations, including the sets of five and six mutations most correlated with improved enrichment ratios, which is why the same antibodies appear in both figures.

### Selected antibody variants display increased affinity and high conformational specificity

We next generated the selected antibodies as Fc-fusion proteins and evaluated their affinities and conformational specificities. To critically evaluate our antibodies, we directly compared them with two clinical-stage antibodies specific for Aβ, namely aducanumab and crenezumab. Aducanumab recognizes an N-terminal Aβ epitope (residues 3–7) and selectively recognizes Aβ fibrils and oligomers relative to disaggregated Aβ (14, 15). In contrast, crenezumab recognizes a central Aβ epitope (residues 13–24) and binds to both aggregated and disaggregated Aβ (14, 15). We grafted the variable domains of each clinical-stage antibody onto an IgG1 scaffold with a human Fc fragment, which resulted in differences in both antibody sequences outside of the variable regions, including in the C_H_1, hinge and Fc regions, between the antibodies tested in this study and the actual clinical-stage antibodies (*e.g.*, crenezumab is an IgG4 antibody). Herein we refer to these antibodies as their common names despite these differences. The selected antibody clones and clinical-stage antibodies both expressed well (purification yield of >30 mg/l) and were isolated with high purity (Fig. S3).

**Figure 5:**
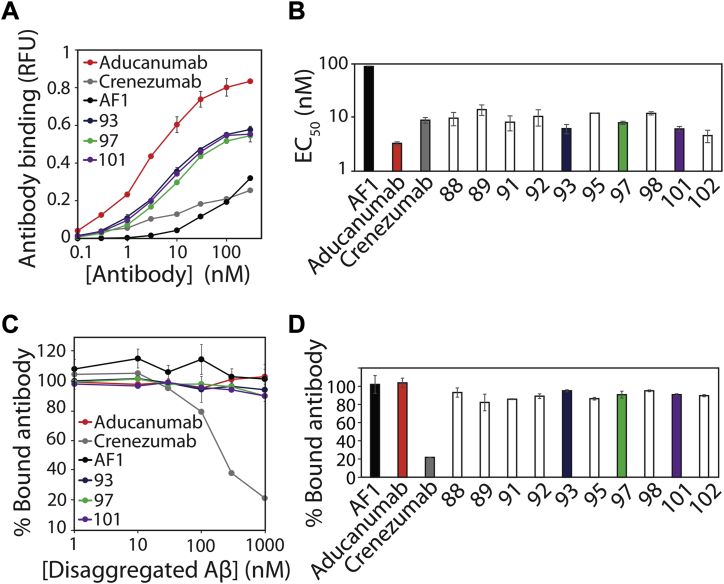
**Identified antibody variants display increased affinity and high conformational specificity for Aβ fibrils.***A*, concentration-dependent binding of selected antibody variants to immobilized Aβ fibrils. *B*, apparent affinity (EC_50_) of selected antibody variants for Aβ fibrils. *C*, binding analysis of antibodies (30 nM) preincubated with different concentrations of disaggregated Aβ prior to binding to immobilized Aβ fibrils. *D*, percentage of bound antibody to Aβ fibrils for antibodies (30 nM) preincubated with disaggregated Aβ (1000 nM). In (*A*–*D*), clinical-stage Aβ antibodies (aducanumab and crenezumab) were used for comparison, the results are average values, and the error bars are standard deviations (two independent repeats).

Given the primary goal of our work to affinity mature our lead Aβ antibody (AF1), we evaluated the apparent affinity of the selected antibody variants relative to AF1 and the clinical-stage antibody controls (Fig. 5, *A* and *B*). As expected, we observed modest affinity for AF1 binding to Aβ fibrils (EC_50_ of 99 ± 2 nM). Notably, we observed significant (order of magnitude) increases in affinity for all of the selected antibody variants, and the EC_50_ values (4–13 nM) were similar to crenezumab (9 ± 1 nM) and modestly higher than aducanumab (3 ± 0.2 nM).

Nevertheless, we have observed that it is relatively common to lose antibody conformational specificity during *in vitro* affinity maturation. Therefore, we next evaluated if the affinity-matured antibodies retained conformational specificity (Fig. 5, *C* and *D*). To evaluate this, we preincubated the antibodies (30 nM) with various concentrations of disaggregated Aβ and then evaluated their fibril-binding activity. As expected, crenezumab displayed low conformational specificity, and its binding to Aβ fibrils was inhibited due to competition with disaggregated Aβ. Conversely, aducanumab binding to fibrils was weakly inhibited by disaggregated Aβ, which is consistent with its high conformational specificity (14). Notably, the binding of our affinity-matured clones to Aβ fibrils was also weakly inhibited by disaggregated Aβ (82–99% bound antibody at 1000 nM disaggregated Aβ) and behaved similar to the parental antibody (AF1).

**Figure 6:**
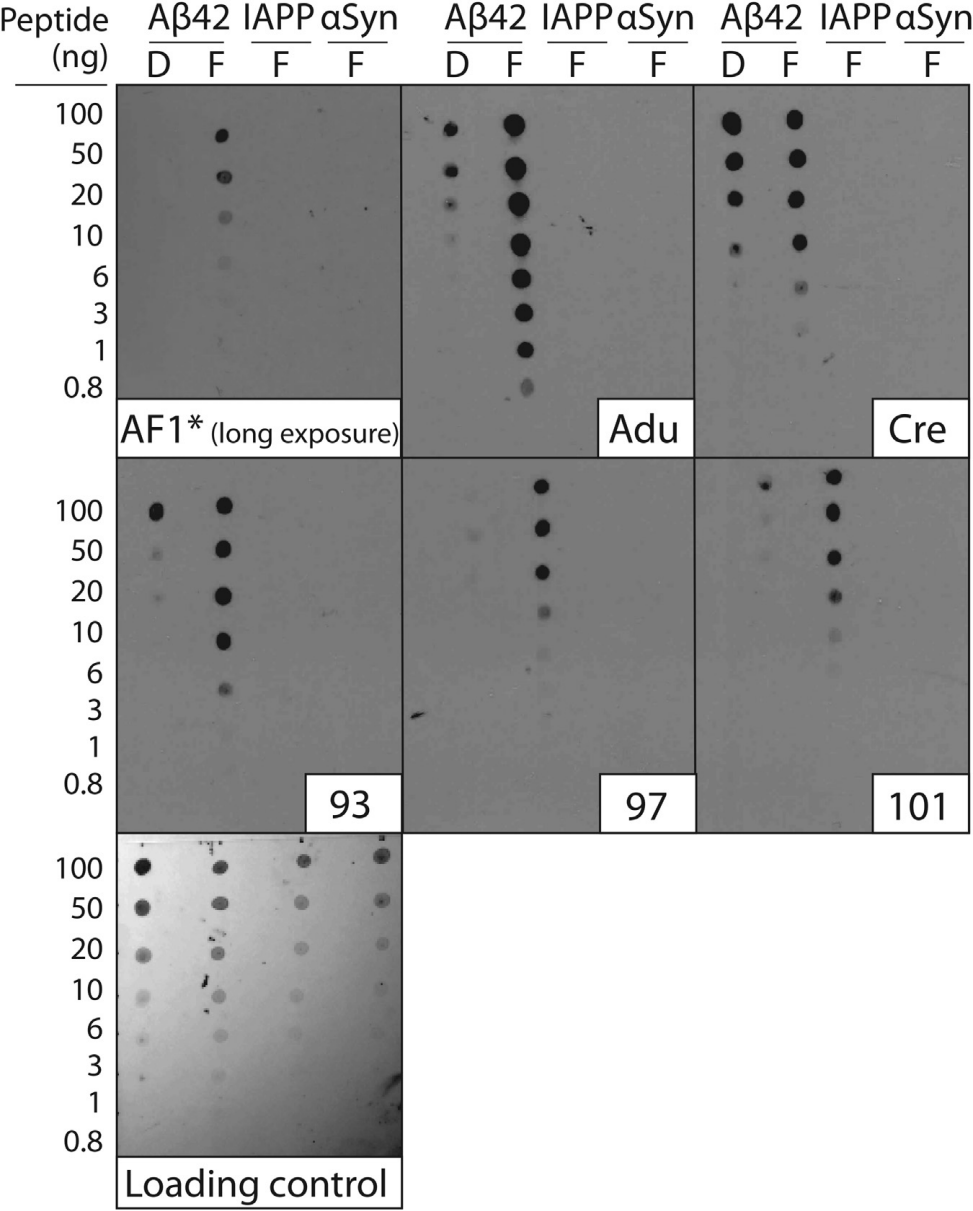
**Immunoblot analysis of the conformational and sequence specificity of the affinity-matured Aβ antibodies.** Fibrillar (F) Aβ, islet amyloid polypeptide (IAPP), α-synuclein (αSyn), and disaggregated (D) Aβ were immobilized on nitrocellulose membranes and probed with Aβ antibodies (10 nM in PBST with 1% milk), including aducanumab (Adu) and crenezumab (Cre). The blots were imaged after relatively short exposure times (30 s) except for AF1 (45 min exposure). A loading control blot was detected using colloidal silver stain. The experiments were repeated three times and a representative example is shown.

These encouraging results led us to evaluate conformational specificity of the selected antibodies using immunodot blots (Fig. 6). The parental antibody (AF1) displayed weak reactivity at 10 nM and required long exposure times (45 min) to detect signals for Aβ fibrils. Conversely, the clinical-stage antibody controls and the affinity-matured variants at the same concentration developed signals rapidly, as evidenced by their results after a short-time (30 s) exposure (Fig. 6). Aducanumab and the selected affinity-matured variants (clones 93, 97, and 101) displayed relatively high conformational specificity. Moreover, crenezumab displayed little conformational specificity, as expected based on our results in Figure 5. Longer exposures (45 min) for the clinical-stage and affinity-matured variants reveal additional binding to both fibrillar and disaggregated Aβ (Fig. S4). The nonlinear nature of the signals generated *via* immunoblots, especially at long exposure times, is difficult to interpret and caution should be exercised when evaluating them.

**Figure 7:**
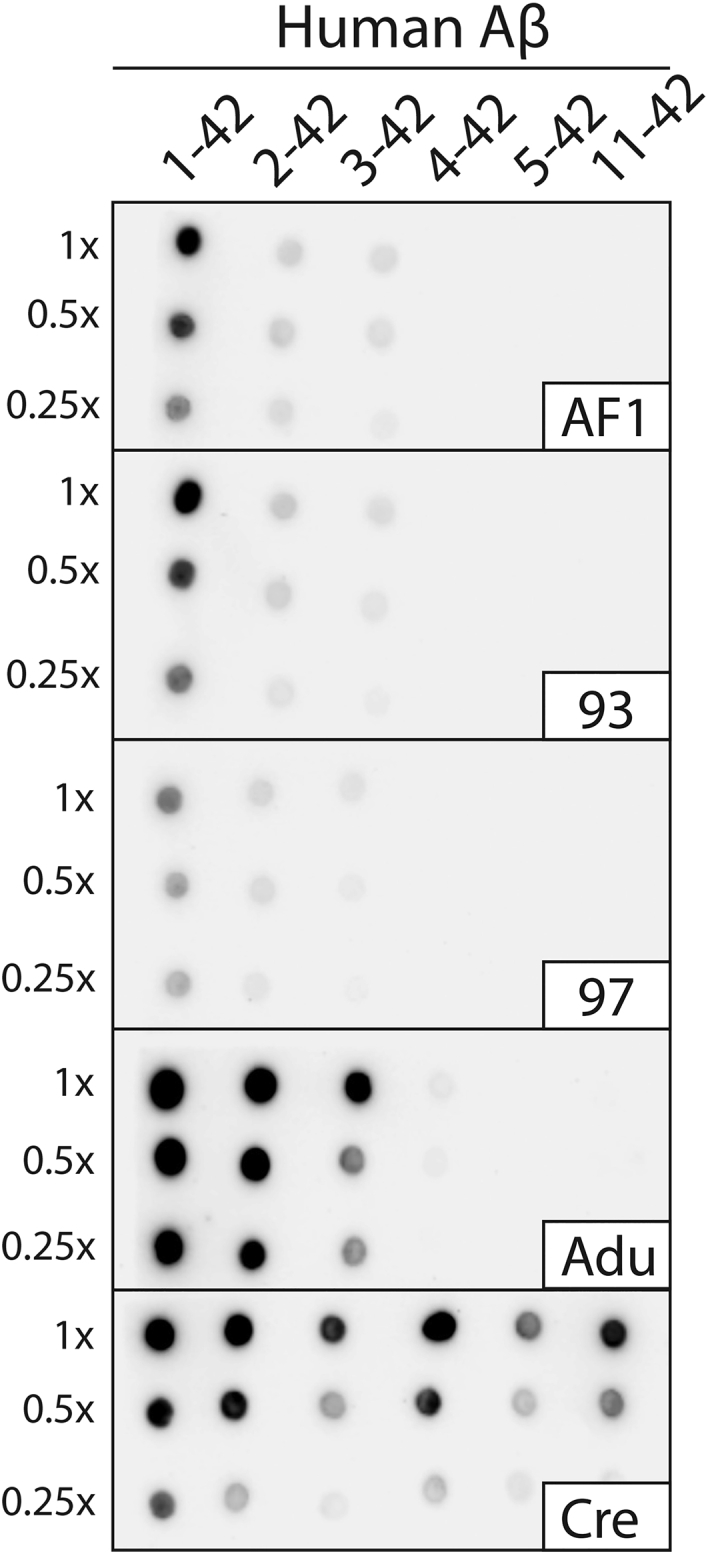
**Conformational epitope analysis of Aβ antibodies.** Aβ fibrils formed with different Aβ peptides, including several N-terminal truncations, were immobilized on nitrocellulose membranes and probed with different Aβ antibodies. Antibody binding was performed overnight at 10 nM in PBST with 1% milk (4 °C). Aducanumab (Adu) and crenezumab (Cre) were included as controls. The image was captured after a 3 min exposure. The experiments were performed three times and a representative image is shown.

Next, we evaluated the epitope recognized by our affinity-matured antibodies relative to aducanumab and crenezumab (Fig. 7). Aβ fibrils that corresponded to full-length Aβ42 or N-terminal truncations were deposited on nitrocellulose blots and probed with various antibodies. AF1 and the affinity-matured clones strongly recognized Aβ1-42 fibrils and weakly recognized fibrils without the first (Aβ2-42) and first two (Aβ3-42) residues. Aducanumab also recognized similar Aβ fibril variants, albeit more strongly, and very weakly recognized Aβ4-42 fibrils. This finding is consistent with the N-terminal epitope of aducanumab reported previously (14, 47). Conversely, crenezumab recognized fibrils of all of the peptide variants (including Aβ4-42, Aβ5-42, and Aβ11–42) given that its epitope is reported to be Aβ residues 13 to 24 (47). These findings demonstrate that the affinity-matured antibodies recognize a conformational epitope involving the Aβ N terminus that is similar to the epitope recognized by aducanumab.

**Figure 8:**
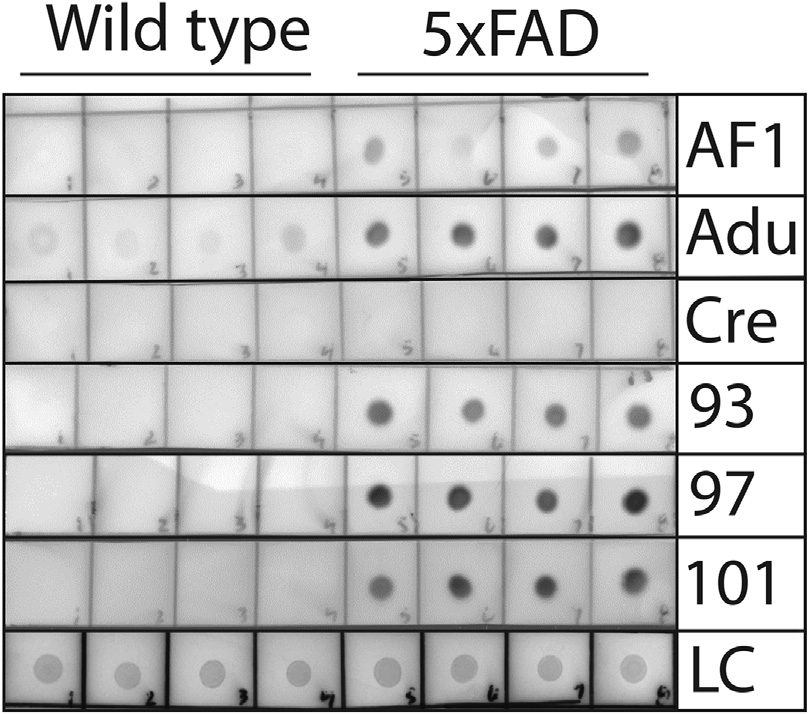
**Immunoblot analysis of transgenic (5xFAD) and wild-type mouse brain samples using Aβ antibodies.** Brain samples (insoluble fraction) obtained from 5xFAD (22–24 months old) and wild-type mice were immobilized on nitrocellulose membranes and probed with Aβ antibodies (50 nM in TBST with 1% milk), including aducanumab (Adu) and crenezumab (Cre). The blots were imaged after a relatively short exposure (15 s). Ponceau S staining was used as a loading control (LC). The experiments were repeated three times and a representative example is shown.

**Figure 9:**
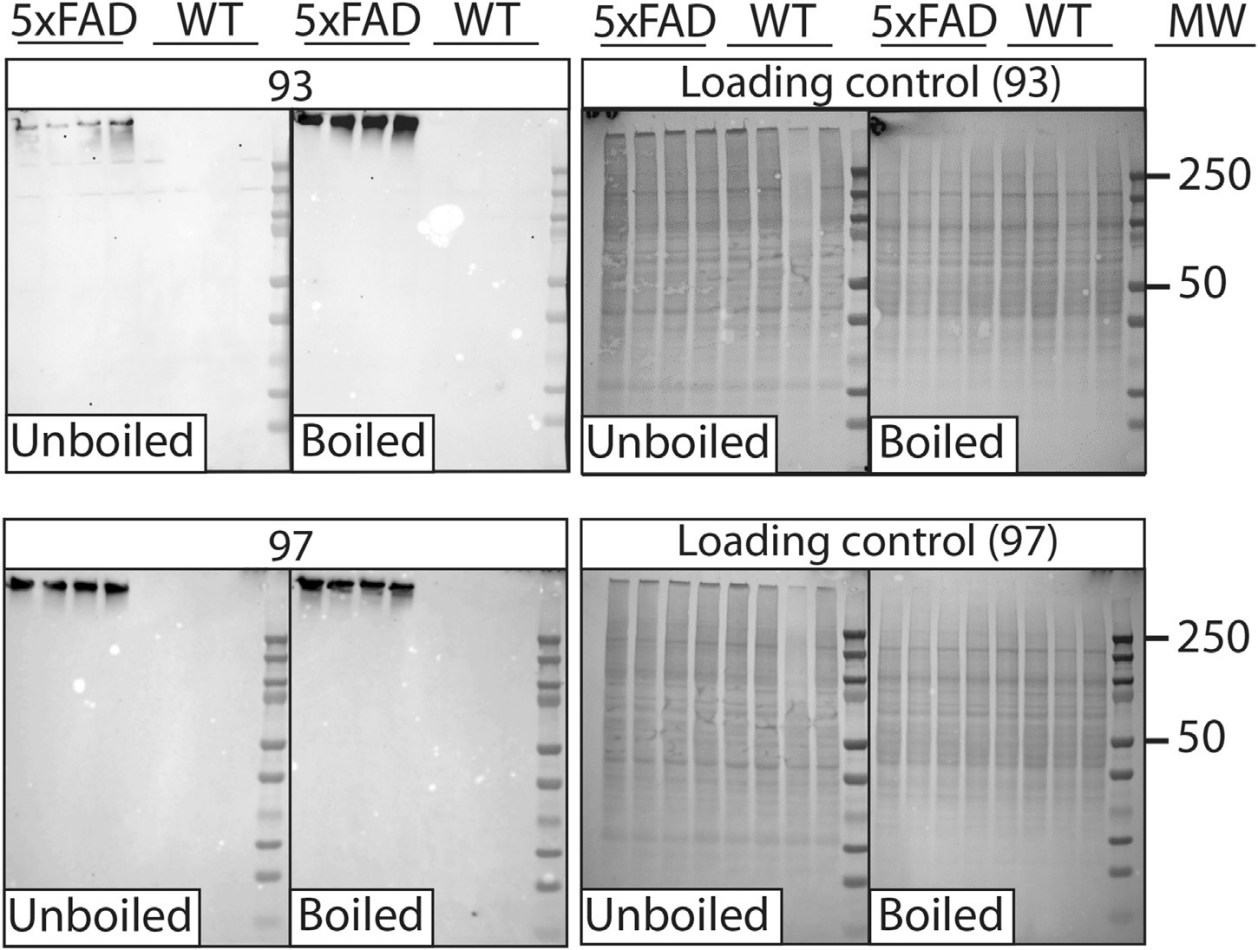
**Western blot analysis of 5xFAD and wild-type mouse brain samples using affinity-matured Aβ antibodies.** Brain samples (PBS insoluble fraction) isolated from 5xFAD (22–24 months old) and wild-type (WT) mice were processed (with or without boiling) via SDS-PAGE, transferred to nitrocellulose membranes, and probed with a subset of Aβ antibodies (100 nM in TBST with 1% milk). The blots were imaged after 6 min of exposure. Ponceau S staining was used as loading control. The experiments were repeated three times and a representative example is shown.

We next evaluated if the affinity-matured antibodies recognize Aβ aggregates formed *in vivo* (Figs. 8 and 9, Figs. S5 and S6). Therefore, we first evaluated the antibodies using immunodot blots of brain homogenates obtained from transgenic mice that overexpress humanized mutant amyloid precursor protein and presenilin 1 (5xFAD) relative to control (wild-type) mice (Fig. 8 and Fig. S5). The parental antibody (AF1) displayed weak immunoreactivity with the 5xFAD samples, while the selected clones (93, 97, and 101) displayed strong and specific detection of 5xFAD samples from four mouse brains relative to those from four control mouse brains. Interestingly, aducanumab detected the 5xFAD samples and also weakly reacted with the wild-type samples, while surprisingly crenezumab failed to detect either type of sample. At longer exposures, aducanumab and crenezumab displayed high background while the affinity-matured antibodies displayed strong and specific recognition of 5xFAD samples (Fig. S5). Moreover, we confirmed these findings for two affinity-matured antibodies (clones 93 and 97) using western blotting and detected strong and specific signals for the 5xFAD samples for the PBS-insoluble (Fig. 9) and PBS-soluble fractions (Fig. S6). For the latter samples, we did not observe antibody binding to low-molecular-weight Aβ species for either the affinity-matured antibody (clone 97) or a sequence-specific antibody (NAB 228) that detects both low and high-molecular-weight Aβ species (23). Finally, we also found that the affinity-matured antibodies recognized Aβ conformers in the human brain-tissue lysates of Alzheimer’s patients *via* immunodot blotting (Fig. S7).

**Figure 10:**
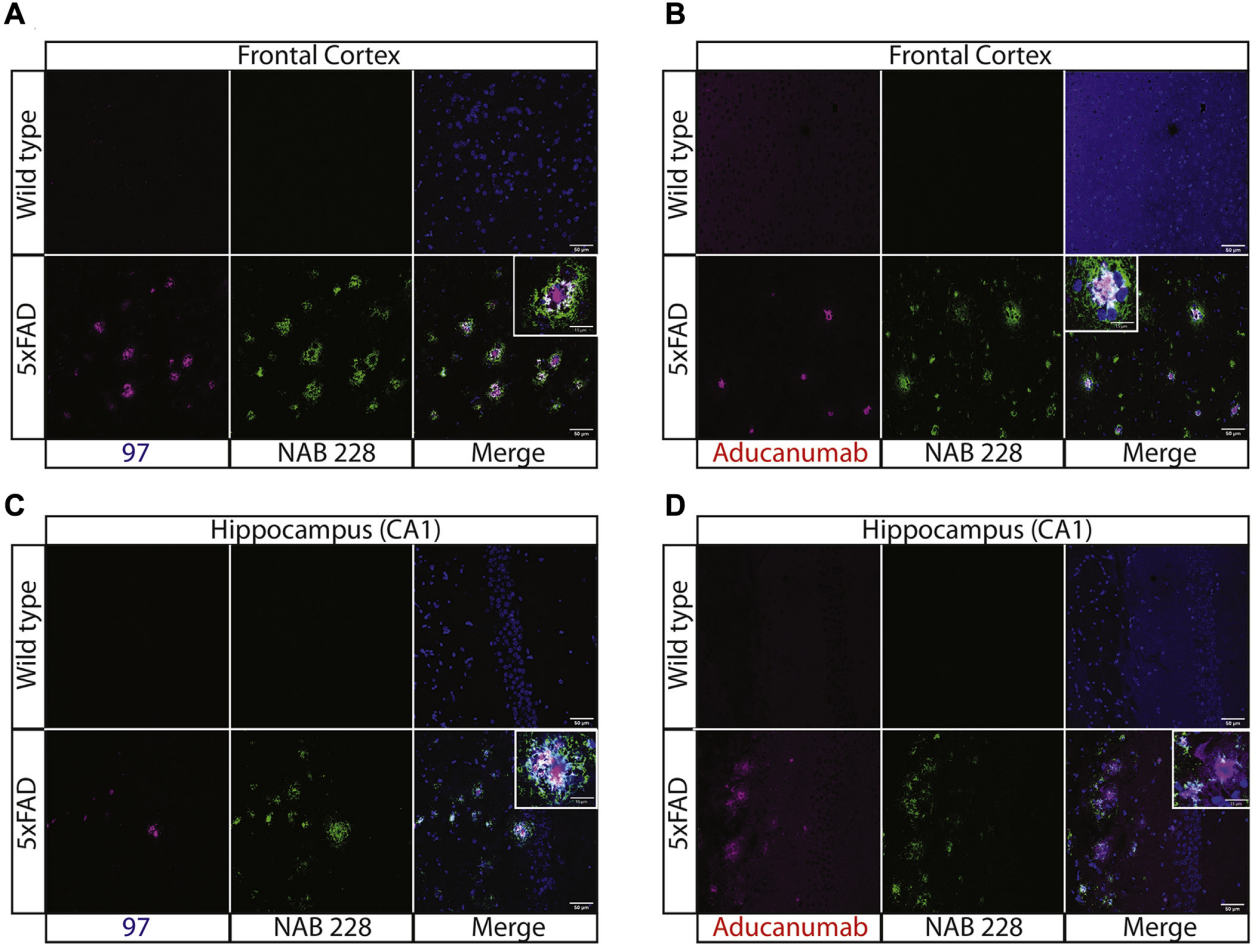
**Immunofluorescence staining of 5xFAD and wild-type mouse brain sections using Aβ antibodies.***A*–*D*, mouse brain sections from the (*A* and *B*) frontal cortex and (*C* and *D*) hippocampus (CA1) were stained using conformational antibodies [clone 97 in (*A*) and (*C*) and aducanumab in (*B*) and (*D*)] at 200 nM, a sequence-specific Aβ antibody (NAB 228; recognizes Aβ1-11) at 1:200x dilution, and DAPI. The 5xFAD mice were 8 months old. Slides were imaged using Leica SP5 confocal microscope. The scale bars are 50 μm for the main images and 15 μm for the inset images.

We also evaluated the ability of the affinity-matured antibodies to stain Aβ aggregates in tissue sections of transgenic (5xFAD) mouse brains relative to wild-type mouse brains (Fig. 10). Clone 97 selectively recognized plaques in the frontal cortex of 5xFAD mouse brains, while a sequence-specific Aβ antibody (NAB 228) recognized more diffuse material that surrounded the plaque cores, as observed by the lack of significant overlap of immunostaining for the two antibodies (Fig. 10*A*). Aducanumab displayed similar staining of Aβ plaques and also displayed little overlap in staining with the sequence-specific antibody (Fig. 10*B*). Notably, aducanumab displayed higher levels of nonspecific binding to wild-type tissue than clone 97. We observed similar patterns of immunostaining for hippocampus (CA1) tissue samples using clone 97 (Fig. 10*C*) and aducanumab (Fig. 10*D*). Overall, these results demonstrate that our affinity-matured antibodies recognize Aβ aggregates formed *in vitro* and *in vivo* with high affinity and conformational specificity and compare favorably to clinical-stage Aβ antibodies.

### Affinity-maturated antibodies display favorable biophysical properties

**Figure 11:**
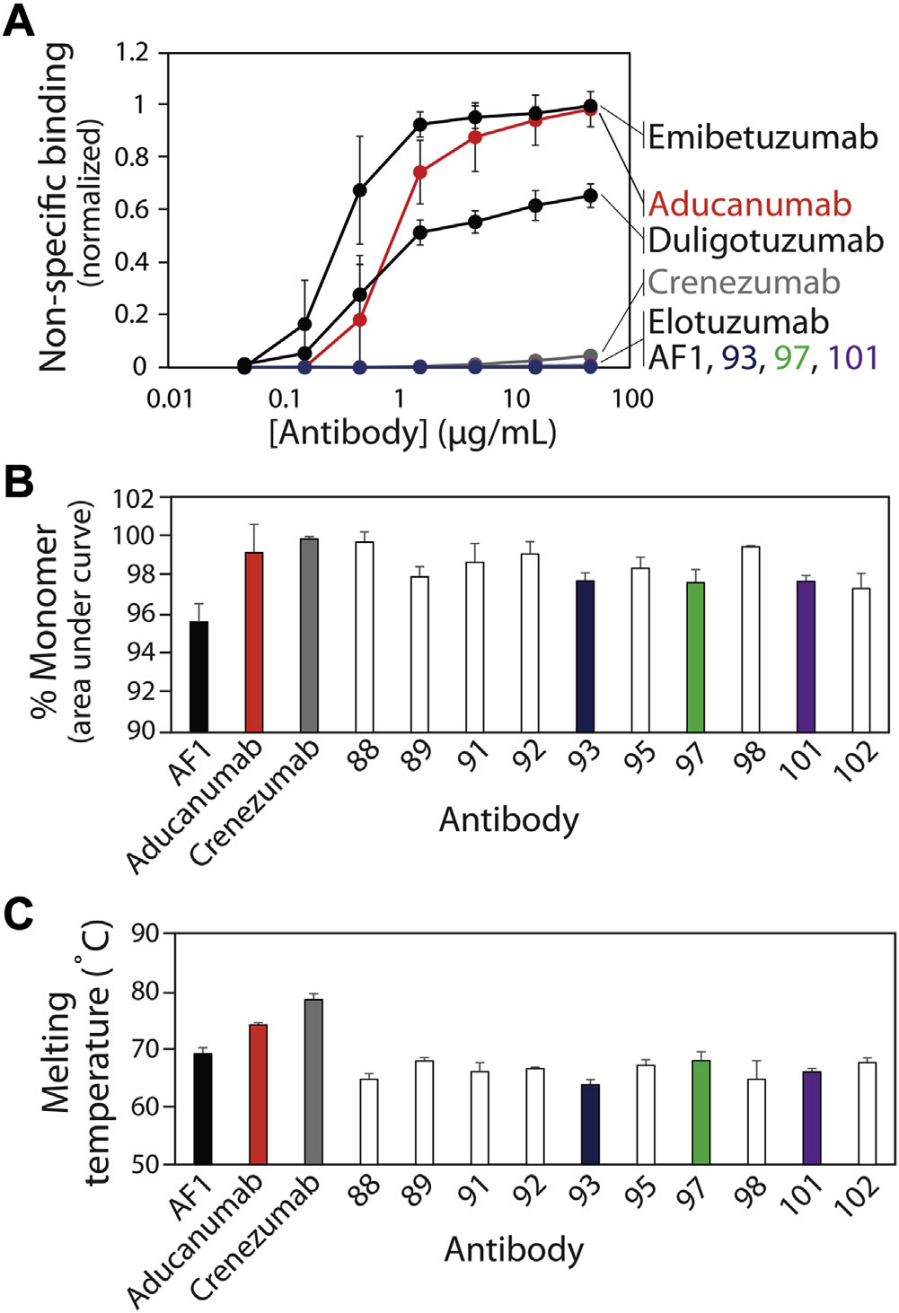
**Biophysical characterization of Aβ antibodies.***A*, antibody nonspecific binding to soluble membrane proteins. The soluble membrane proteins were biotinylated and their binding to immobilized antibodies was evaluated *via* flow cytometry. *B*, percentage of monomeric antibody evaluated *via* size-exclusion chromatography. *C*, antibody melting temperature (midpoint of unfolding) evaluated using dynamic scanning fluorimetry. In (*A*–*C*), the values are averages and the error bars are standard deviations (three independent repeats).

One of the most common limitations of using *in vitro* antibody discovery and engineering methods is the generation of antibodies with suboptimal biophysical properties – such as low stabilities, solubilities, and specificities – relative to antibodies generated by the immune system (36, 48, 49, 50). Therefore, we next sought to evaluate the biophysical properties of our affinity-matured antibodies to determine if they maintained favorable specificities and stabilities (Fig. 11). First, we evaluated nonspecific binding for our antibodies using a previously reported polyspecificity reagent (PSR) that is composed of soluble membrane proteins isolated from CHO cells (Fig. 11*A*) (36, 51). Antibody binding to this reagent is a strong indicator of the level of antibody specificity and the likelihood of abnormal pharmacokinetics (52). Encouragingly, our affinity-matured antibodies displayed extremely low levels of nonspecific interactions that were similar to their parental antibody (AF1) and a control clinical-stage antibody with high specificity (elotuzumab) (36). Moreover, the matured antibodies were even more specific than crenezumab, which also displayed relatively low levels of nonspecific binding. Interestingly, aducanumab displayed much higher levels of nonspecific binding that were similar to the control clinical-stage antibodies with high levels of nonspecific binding (emibetuzumab and duligotuzumab) (36). Although these results were performed using the affinity-matured antibodies after only one-step purification (Protein A) and the control clinical-stage antibodies after two-step purification (Protein A and size-exclusion chromatography (SEC)), we obtained similar nonspecific binding measurements for the former antibodies after two-step purification (Fig. S8)

We also evaluated the physical stabilities of our antibodies (Fig. 11, *B* and *C*). Antibodies with poor stability often display aggregation at low pH during elution from Protein A columns (53, 54, 55, 56, 57, 58). Therefore, we evaluated the percentage of monomeric antibody after Protein A purification for the affinity-matured antibodies relative to the control clinical-stage antibodies (Fig. 11*B* and Fig. S9). Encouragingly, we observed that the affinity-matured antibodies displayed high levels of monomeric protein (>95%) that were similar to the clinical-stage antibodies. Moreover, we evaluated the melting temperatures of our single-chain antibodies (as scFv-Fc fusion proteins) relative to the clinical-stage IgGs (Fig. 11*C* and Fig. S10) to evaluate if affinity maturation reduced stability (59, 60, 61). Due to the lack of constant (C_H_1 and C_L_) domains, it is expected that the single-chain antibodies will have lower stabilities than the clinical-stage IgGs. Nevertheless, we find that the affinity-matured antibodies displayed high stabilities (*T*_*m*_ values of 64–69 °C) that were comparable to the parental antibody (AF1, *T*_*m*_ of 69 °C) and modestly lower than the clinical-stage IgGs (74–79 °C). In summary, our affinity-matured antibodies display a combination of biophysical properties that are favorable and unique in comparison to clinical-stage Aβ antibodies.

### Additional affinity maturation does not compromise conformational and sequence specificity

We evaluated the feasibility of using our methods to further affinity mature one of the best antibody variants (clone 97) while maintaining high conformational specificity and low nonspecific binding. Therefore, we designed and screened a sublibrary for clone 97 with mutations in heavy chain CDR1 and light chain CDR2, as these two CDRs were the only ones not mutated during the initial round of discovery (heavy chain CDR3) and the first round of affinity maturation (heavy chain CDR2 and light chains CDRs 1 and 3).

**Figure 12:**
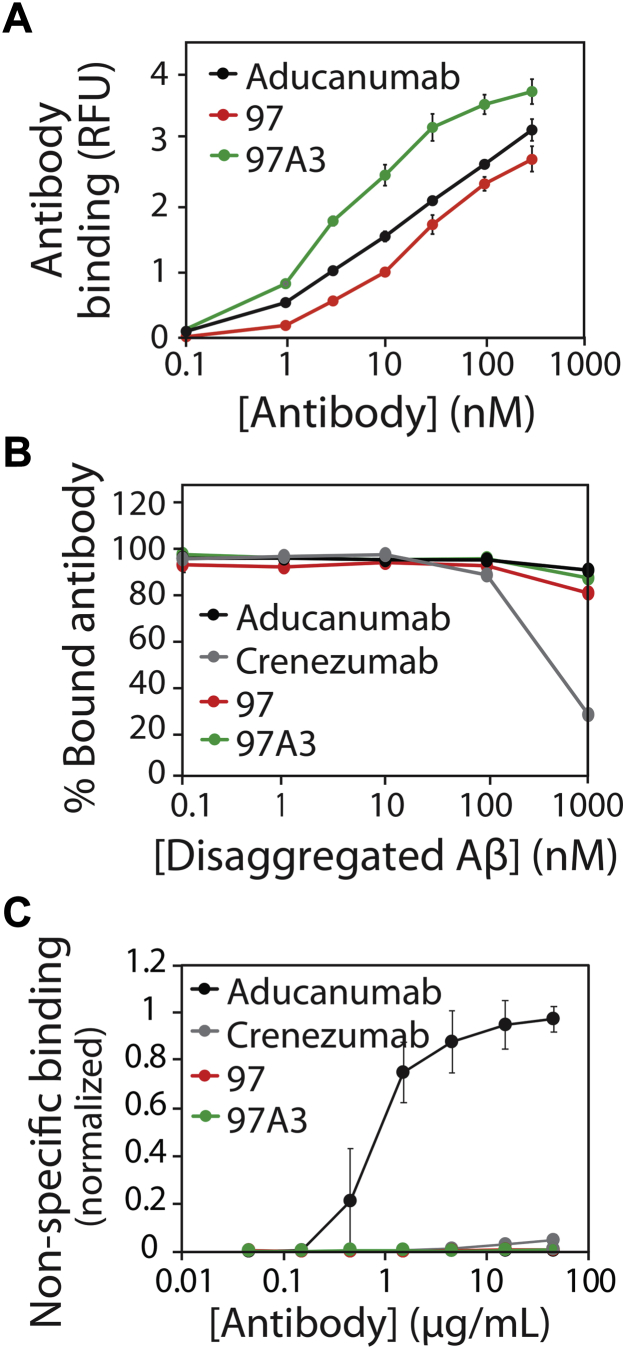
**Additional affinity maturation results in an Aβ antibody variant (97A3) with improved affinity, high conformational specificity, and low non-specific binding.***A*, concentration-dependent binding of clone 97A3 to Aβ fibrils relative to its parental antibody (clone 97) and aducanumab. *B*, binding analysis of antibodies (30 nM) preincubated with different concentrations of disaggregated Aβ prior to binding to immobilized Aβ fibrils. *C*, antibody nonspecific binding to soluble membrane proteins. In (*A*) and (*B*), the experiments were performed as described in Figure 5. In (*C*), the experiments were performed as described in Figure 11.

MACS selections against Aβ42 fibrils yielded a single enriched antibody variant with five mutations in light-chain CDR2 (97A3; Fig. 12). Notably, this clone displayed higher apparent affinity than the parental antibody (∼sixfold improvement) and aducanumab (∼threefold improvement; Fig. 12*A*). Given that different batches of fibrils were used to perform the binding experiments in Figures 5 and 12, the EC_50_ values for clone 97 (8 ± 1 nM in Fig. 5 and 18 ± 1 nM in Fig. 12) and aducanumab (3 ± 1 nM in Fig. 5 and 10 ± 2 nM in Fig. 12) were modestly different. Moreover, the affinity-matured antibody (97A3) displayed high conformational specificity that was similar to clone 97 and aducanumab (Fig. 12*B*) and low nonspecific binding that was similar to clone 97 and much lower than aducanumab (Fig. 12*C*). Moreover, 97A3 was mostly monomeric after one-step Protein A purification (>93%) and displayed high stability (*T*_*m*_ of 69 °C ± 0.5 °C) that was similar to the parental antibody (97% monomer and *T*_*m*_ of 68 °C ± 2 °C; Fig. S11). This demonstrates that our affinity maturation methods can be used to generate antibodies with superior affinities and levels of nonspecific binding relative to aducanumab while maintaining high conformational specificity and thermal stability.

## Discussion

We have demonstrated a rational and systematic approach for affinity maturing conformational antibodies specific for insoluble polypeptide aggregates. Prior to this work, we were skeptical about the feasibility of this process due to the likelihood of strong avidity effects between multivalent yeast-displayed antibodies and multivalent Aβ aggregates immobilized on magnetic beads. In the case of soluble and monovalent antigens, it is much easier to select affinity-matured antibodies using yeast surface display because of the reduced antigen-specific avidity effects and the ability to use FACS. However, in the case of insoluble aggregates, it is typically not possible to use FACS because of the particulate and insoluble nature of polypeptide aggregates.

While any antibody engineering campaign has the potential to succeed if enough clones are screened, we found a surprisingly high level of success at identifying affinity-matured clones using our reported approach. For example, all 19 of the clones that were identified *via* our deep sequencing analysis displayed increased affinity during our primary screens performed with immunodot blots. Moreover, all of the 15 clones tested for conformational specificity displayed low levels of binding to disaggregated Aβ in our competition experiments (Fig. 5, *C* and *D*). Finally, all of the 15 clones tested for Aβ fibril affinity displayed 8- to 20-fold improvements in their EC_50_ values compared with AF1 (Fig. 5, *A* and *B*).

Given this higher-than-expected success rate, this raises the question of why this approach was successful and what are the most important aspects of this methodology to consider for future studies. One potentially relevant observation is related to how we identified sets of mutations most correlated with improved enrichment ratios using deep sequencing. This process assumes that the sets of mutations (*e.g.*, sets of six mutations) govern the improved behavior and ignores the residues at the other randomized sites. It is logical that introducing these sets of mutations into the parental antibody – without introducing any mutations at the other sites – may improve antibody affinity. However, we found that this approach was much less robust, as <50% (7 out of 15) of the antibody mutants tested using this strategy showed increased affinity (as judged by immunoblots; data not shown). This suggests that mutated residues at sites not considered in a given mutational set (*e.g.*, sites 1 and 2 when evaluating sets of mutations at sites 3–8) contribute to the overall binding activity and were important to our success in identifying affinity-matured variants.

We also suspect that our strategy for designing sublibraries with particular types of mutations contributed to the success of selecting antibody variants with improved affinity while maintaining both conformational specificity for Aβ aggregates and low levels of off-target binding. Given the acidic nature of Aβ42 (theoretical pI of 5.3), it is common in our experience to select positively charged mutations that increase antibody affinity due to attractive electrostatic interactions (40, 61). However, overenrichment in positively charged residues in antibody CDRs is a key risk factor for off-target binding (38, 42, 43, 45, 46, 62, 63, 64, 65). Therefore, we eliminated positively charged mutations from our library design. We speculate that this may have reduced (at least partially) the strong avidity effects due to reduction of relatively long-range (attractive) electrostatic interactions during library sorting. While positive charge is obviously not deleterious in all cases for specific and high-affinity binding, it may be that eliminating positively charged mutations reduces nonspecific electrostatic interactions that frustrate selection of antibody clones with intrinsic increases in affinity due to avidity effects.

It is also notable that our parental (AF1) and affinity-matured antibodies display unusually low levels of nonspecific binding. The origin of the high nonspecific binding for Aβ (aducanumab) and other non-Aβ (emibetuzumab and duligotuzumab) clinical-stage antibodies relative to low nonspecific binding for Aβ (crenezumab) and non-Aβ (elotuzumab) clinical-stage antibodies appears linked to the charge properties of the antibody variable regions (Table S1). The three antibodies with high nonspecific binding have variable fragments (Fvs) that are either strongly positively charged (+9.1 for aducanumab and +5.2 for emibetuzumab) or strongly negatively charged (−4.9 for duligotuzumab), as judged by their theoretical net charges at pH 7.4. In contrast, the antibodies with low nonspecific binding have near neutrally charged Fvs (+0.2 for crenezumab and −0.9 for elotuzumab at pH 7.4). Moreover, AF1 and the affinity-matured clones with low levels of nonspecific interactions have weakly positively charged Fvs (+0.2 for AF1 and +1.2 to +2.2 for the first generation affinity-matured variants and +2.2 for the second generation variant) that are intermediate to the antibodies with low and high levels of nonspecific interactions. This suggests that near neutrally (or weakly positively) charged Fvs may be optimal for high antibody specificity. This is consistent with the fact that clinical-stage antibodies with low levels of nonspecific and self-interactions typically have Fvs with near neutral charges (1.5 ± 2.5 at pH 7.4) (41), which overlaps with the observed Fv charges for the antibodies with high specificity in this study.

Aβ-specific antibodies typically have more positively charged Fv regions (theoretical net charge at pH 7.4) than antibodies in this study with high specificity, including gantenerumab (+6.1), ponezumab (+4.2), BAN2401 (+2.3), and solanezumab (+3.2) in addition to aducanumab (+9.1; Table S1). The acidic nature of Aβ, as noted above, is likely one reason for this bias toward positively charged antigen-binding sites. However, in the case of Aβ antibodies such as aducanumab that have abnormally positively charged Fv regions, it is possible that these properties are linked to improved transport across the blood–brain barrier. A key step in adsorptive-mediated transcytosis – which can be specific [receptor-mediated (66, 67, 68, 69)] or nonspecific [electrostatically mediated (70)] – is antibody binding at the cell surface. Antibodies with positively charged Fvs are known to interact with negatively charged cell membranes and display enhanced cellular uptake (62, 70, 71). Moreover, antibodies that display high levels of nonspecific interactions are linked to increased transcytosis in cell culture (69). Therefore, we speculate that the positively charged properties of aducanumab variable regions – while potentially deleterious in terms of off-target binding – may be beneficial in promoting cellular internalization and transcytosis.

However, it is also notable that administration of Aβ antibodies such as aducanumab and bapineuzumab has been linked to amyloid-related imaging abnormalities (ARIA) detected by magnetic resonance imaging (15, 47, 72, 73, 74). ARIA is associated with disruption of the blood–brain barrier (regional vasogenic edema). Interestingly, the effectiveness of aducanumab at reducing amyloid in the brain is associated with increased frequency of brain edema or ARIA (15). This may suggest that the nonspecific mechanism by which antibodies such as aducanumab enter the brain – which likely is enhanced by positively charged Fv regions – results in a narrow therapeutic index (68). This also suggests that using bispecific antibodies that combine more specific Aβ antibodies – such as those reported in this study – with antibodies that target receptors at the blood–brain barrier (75, 76, 77, 78) may enable the use of lower antibody doses and be a safer and more effective strategy for targeting Aβ aggregates in the brain.

It is also important to consider several other aspects of our methods and findings. First, we evaluated the apparent affinities (EC_50_ values) of the antibodies at relatively low antigen concentrations (1% biotinylated fibrils immobilized on beads at 1 μM), which we found to be important to differentiate between the parental (AF1) and affinity-matured variants. At higher antigen concentrations (10% biotinylated fibrils immobilized on beads at 6 μM), we observed smaller improvements for the affinity-matured variants (data not shown), which is likely due to avidity effects. Second, we evaluated the immunodot blots at different exposure times using X-ray film (Fig. 6 and Fig. S4) and found that the apparent conformational specificities were dependent on exposure time. Caution should be exercised when evaluating antibody conformational specificity using dot blots because the signal for aggregates in some cases can readily saturate while the signal for disaggregated peptide can continue to increase with exposure time, leading to potentially misleading results. Third, our deep sequencing analysis only scratched the surface of the many promising antibody candidates that could be evaluated in the future. Due to errors in our initial evaluation of the deep sequencing data, the reported antibody variants have favorable but not the most favorable sets of mutations and corresponding enrichment ratios. This suggests that there may be additional opportunities to generate even better antibodies using this approach in the future.

Our findings suggest a number of additional future directions. First, our affinity maturation methodology could be readily applied to further increase the affinities of the reported Aβ antibodies in this study. Second, we expect that this approach could be applied to evolve not only the affinity but also the conformational specificity of existing antibodies against diverse types of amyloidogenic aggregates. The ability to control antigen presentation to antibody sublibraries enables the selection of variants with increased conformational specificity in addition to increased affinity. This is particularly important for aggregates such as prefibrillar oligomers that are challenging to isolate, stabilize, and use as antigens for immunization. Moreover, even for conformational antibodies discovered by immunization, it is likely that additional affinity maturation would be beneficial for their use in diagnostic and therapeutic applications. Indeed, we are currently testing the generality of these methods for maturing the affinity and conformational specificity of antibodies specific for a number of different amyloidogenic proteins.

## Experimental procedures

### Aβ solubilization and fibril preparation

Aβ fibrils were prepared as described previously (23). Lyophilized Aβ1-42 (Anaspec, AS20276) and biotinylated Aβ1-42 (Anaspec, AS23526-05) peptides were dissolved in hexafluoro-2-isopropanol (HFIP), aliquoted, and stored at −80 °C at 1 mg/ml (Aβ1-42) and 0.17 mg/ml (biotinylated Aβ1-42). For fibril preparation, aliquots were thawed and HFIP was evaporated overnight. Peptides were dissolved in 50 mM NaOH and ultracentrifuged at 221,000*g* at 4 °C for 1 h. The supernatant (typically 45 μl) was collected, transferred to a new tube, and neutralized with nine times the volume (typically 405 μl) of acidified PBS (PBS with 4.7 mM HCl). The peptide concentration was determined by measuring the absorbance at 280 nm.

Unlabeled fibrils were assembled at 37 °C for at least 3 days without agitation by further diluting the soluble peptide in PBS to a final concentration of 12.5 μM along with the addition of 10% fibril seeds (1.25 μM of preformed fibrils). Biotinylated fibrils were assembled in similar manner except that the assemblies were doped with 1 or 10% biotinylated Aβ monomer (final concentration of Aβ monomer was 12.5 μM). After at least 3 days, the assemblies were ultracentrifuged at 221,000*g* for 1 h (4 °C). The supernatant was discarded and the fibril pellet was resuspended in fresh PBS (typically ∼100 μl for unlabeled fibrils). For biotinylated fibrils, the pellet was resuspended in the same initial volume to achieve a nominal fibril concentration of 12.5 μM. Unlabeled fibrils were briefly sonicated for 30 s (three cycles of 10 s on and 30 s off) on ice and their concentration was determined by the BCA assay. Biotinylated fibrils were sonicated for 2 min (12 cycles of 10 s on, 30 s off) on ice before incubating them with Streptavidin Dynabeads (Invitrogen, A11047). For fibril bead preparation for sorting, 10% biotinylated fibrils (6 μM) were mixed with 10^7^ beads in a final volume of 400 μl in PBSB (PBS with 1 mg/ml BSA). For fibril bead preparation for antibody analysis, 1% biotinylated fibrils (1 μM) were mixed with 10^7^ beads in a final volume of 400 μl in PBSB.

### Antibody library generation

Antibody library genes (theoretical diversity of 1.1 × 10^8^) were prepared by PCR. Three degenerate oligos were designed with diversity in LCDR1, LCDR3, and HCDR2. Four individual PCRs were performed for the AF1 scFv gene using the yeast surface display plasmid (23) as a template, three of which used degenerate primers. Overlap PCR was then performed to combine DNA fragments with terminal primers. The PCR product was purified *via* a 1% agarose gel followed by gel extraction (Qiagen, 28706). The wild-type AF1 scFv plasmid was double digested with NheI-HF (New England Biolabs, R3131L) and XhoI (New England Biolabs, R1046L), treated with alkaline phosphatase (New England Biolabs, M0525L), and purified *via* a 1% agarose gel. The digested backbone was cut and purified with a gel extraction kit. The scFv gene and digested backbone were ligated by homologous recombination in the EBY100 yeast strain (*Saccharomyces cerevisiae*) *via* electroporation, as described earlier (23, 79). The total number of transformants obtained was ∼10^9^.

For clone 97 affinity maturation, a library was constructed by diversifying five positions in light-chain CDR2 and five positions in heavy-chain CDR1 using NNK codons. The antibody genes were prepared by overlap extension PCR. The plasmid backbone was digested with NheI-HF and XhoI, treated with alkaline phosphatase, and purified by 1% agarose gel. The scFv antibody library genes were ligated by homologous recombination in the yeast strain EBY100 *via* electroporation as described above. The total number of transformants obtained was ∼5 × 10^8^.

### Yeast surface display and sorting

Five rounds of magnetic-activated cell sorting (MACS) were performed against Aβ fibrils (10% biotinylated fibrils) immobilized on streptavidin beads. For round 1, yeast cells (10^9^) expressing antibodies were sorted first using negative selections (three times) against disaggregated (biotinylated) Aβ immobilized on streptavidin beads (10^7^ beads per round) in PBSB, as described previously (23). Next, the remaining yeast cells after negative selections were sorted against 10^7^ beads coated with Aβ fibrils in PBSB supplemented with 1% milk for 3 h (room temperature). The yeast cells bound to fibril-coated beads were collected by magnetic separation, washed, and grown in low pH SD-CAA media (20 g/l of dextrose, 6.7 g/l of yeast nitrogen base without amino acids, 5 g/l of casamino acids, 16.75 g/l of sodium citrate trihydrate, 4 g/l citric acid). Dilutions were plated to estimate the number of cells collected for the selections against Aβ fibrils. For round 2, the sorting was performed in similar way except with a reduced number of yeast cells (10^7^ cells).

For rounds 3, 4, and 5, the sorting was performed in a similar way as round 2 except that the negative selections were performed against IAPP fibrils (10% biotinylated IAPP fibrils immobilized at a peptide concentration of 6 μM). IAPP and biotinylated IAPP peptide were dissolved in HFIP at 1 mg/ml, aliquoted, and frozen at −80 °C. Next, the peptides were thawed, followed by snap freezing in liquid nitrogen and lyophilization. The lyophilized peptide was dissolved at pH 7.4 in 20 mM Tris (typically 150 μl) and centrifuged at 21,000*g* for 10 min to remove aggregates. The supernatant (typically 145 μl) was then transferred to a new tube. The peptide concentration was determined by measuring the absorbance at 280 nm. Fibrils were assembled at 32 μM (10% biotinylated peptide) at 37 °C and 300 RPM for 3 to 4 days. Post assembly, fibrils were purified by ultracentrifugation at 221,000*g* for 1 h at 4 °C. The fibril pellet was resuspended to the same final volume to achieve fibrils at 32 μM. For bead preparation, fibrils were sonicated for 2 min (10 s on, 30 s off) on ice followed by mixing with streptavidin beads (6 μM fibrils with 10^7^ beads in a final volume of 400 μl)

For clone 97 affinity maturation, MACS was performed as described above. For round 1, 10^9^ yeast cells expressing antibodies were incubated with 10^7^ beads coated with Aβ fibrils (1% biotinylated fibrils were immobilized at 1 μM Aβ42) at room temperature for 3 h in PBSB with 1% milk. Yeast cells bound to fibril-coated beads were collected *via* a magnet, washed once with ice-cold PBSB, and grown in SDCAA media. For rounds 2 and 3, sorting was performed in a similar way except with 10^7^ cells. In round 4, a negative selection was performed against biotinylated and disaggregated Aβ monomer (1000 nM) *via* FACS. Antibody display was detected using mouse anti-myc antibody (Cell Signaling, 2276S) at 1/1000x dilution followed by secondary staining with goat anti-mouse IgG (H + L) AF488 (Invitrogen, A11001) at a 200x dilution. Disaggregated Aβ binding was detected using streptavidin AF647 (Invitrogen, S32357) at 1000x dilution. Yeast cells displaying antibody but not binding to disaggregated Aβ were collected and grown in SDCAA media. For rounds 5, 6, 7, and 8, MACS was performed as described above with 10^7^ cells and 10^7^ beads. In rounds 6, 7, and 8, after incubating yeast with fibril-coated beads, yeast cells bound to such beads were washed (3x for 20 min per wash with end-over-end mixing) with PBSB supplemented with 0.05% Tween 20 to select for antibodies with increased affinity and potentially with lower off-rates.

### Deep sequencing and data analysis

Yeast plasmids containing scFv genes were extracted after regrowing the sorted antibody libraries from rounds 2 to 5 using a Zymoprep Yeast Plasmid Miniprep II Kit (Zymo Research, D2004). PCR was used to amplify a portion of the scFv gene containing LCDR1, LCDR3, and HCDR2 and to add Illumina adapter regions as well as DNA barcodes. These PCR products were run on 1% agarose gels and purified using a QIAquick Gel Extraction Kit (Qiagen, 28704). A second PCR was performed with 2 μl of the purified products using primers that anneal to the Illumina adapter regions. This product was also purified *via* a gel extraction kit. The samples were sequenced using Illumina MiSeq with 300 bp paired-end sequencing reactions.

To analyze the paired-end output fastq files, the two fastq files corresponding to each sample were merged into one fastq file using BBMerge with the qtrim parameter set to 15 (80). The resulting file was converted to a fasta file and each line was analyzed. The lines containing sequences were checked to ensure correct lengths (540 bp) and absence of bases called as “N.” Next, sequences were translated using BioPython (81). If the resulting translations did not contain stop codons and started with the correct amino acid (T), they were further analyzed. Otherwise, the reverse complements of the sequences were translated and checked for the starting amino acids and stop codons. Next, the 11 residues with potential mutations in the sequences were identified and added to a dictionary if they were previously unobserved or increased their count of observation. This process was repeated for every sample and the results were recorded in a csv file.

To select clones for experimental evaluation, mutational analysis was performed to identify sets of mutations most strongly correlated with improved antibody binding. For example, for a given set of potential mutations (*e.g.*, D61G in HCDR2 and D28N, N30Y and A34T in LCDR1), clones were collected that contain those mutations (potentially among others) as well as all the clones with wild-type residues in those positions (irrespective of other mutations). Next, the Spearman correlation coefficients were calculated for the correlations between the enrichment ratios of the identified clones (x-axis) and the frequencies of mutations (y-axis). Mutational analysis was conducted for one to nine mutations, and at least ten clones were required in each of the mutant and wild-type sets. Moreover, the Spearman correlation coefficients were required to be statistically significant (*p*-value < 0.05).

### Mammalian plasmid cloning, expression and purification

Antibody sequences selected from deep sequencing analysis were ordered as separate V_L_ and V_H_ geneblocks. The geneblocks were combined by overlap PCR with primers containing NheI (forward primer) and HindIII (reverse primer) restriction sites. The PCR products were run on 1% agarose gels and purified *via* a Qiagen gel extraction kit. The purified DNA fragments were then double digested by NheI-HF (New England Biolabs, R3131L) and HindIII-HF (New England Biolabs, R3104L) and further purified using a PCR clean-up kit (Qiagen, 28104). HEK293-6E mammalian expression plasmids were double digested with NheI-HF and HindIII-HF followed by alkaline phosphatase treatment. The digested backbone was then gel purified using a 1% agarose gel. DNA inserts and plasmid backbones were ligated by T4 DNA ligase (New England Biolabs, M0202L), and the ligation mixtures were transformed into competent DH5α cells and plated on LB agar plates supplemented with 100 μg/ml ampicillin. Single colonies were picked, grown in LB supplemented with ampicillin, mini-prepped (Qiagen, 27106), and sequence confirmed.

For antibody expression, plasmids (15 μg) were mixed with PEI (45 μg) in F17 media (Invitrogen, A1383502) and incubated at room temperature for 10 to 20 min after vortexing briefly. The resulting mixtures were then added to cells growing in F17 media supplemented with L-glutamine (Gibco, 25030081), Kolliphor (Fisher, NC0917244), and antibiotic G418 (Gibco, 10131035). Yeastolate (BD Sciences, 292804) was added at 20% w/v after 24 to 48 h. The expressions were continued for 4 to 5 days, and media was collected by centrifuging cells at 3500*g* for 40 min. The media was transferred to a new tube and 1 ml of Protein A resin (Pierce, 20333) was added. Media and beads were rocked gently overnight at 4 °C. The beads were collected by passing media through a filter column (Thermo Fisher Scientific, 89898) under vacuum. Beads were washed with 50 to 100 ml of PBS and protein was eluted from the beads in 0.1 M glycine (pH 3). Protein was then buffer exchanged into 20 mM acetate (pH 5) using Zeba desalting column (Thermo Fisher Scientific, 89894), passed through 0.2 μm filters (EMD Millipore, SLGV004SL), aliquoted, and stored at −80 °C. Protein concentrations were determined by measuring the absorbance at 280 nm, and purity was evaluated by SDS-PAGE (Invitrogen, WG1203BOX).

### Analytical size-exclusion chromatography

The purity of antibodies after the first purification step (Protein A) was also evaluated using SEC. A Shimadzu Prominence HPLC System was used that was outfitted with an LC-20AT pump, SIL-20AC autosampler, and FRC-10A fraction collector. Antibodies in 20 mM acetate (pH 5) were buffer exchanged into PBS (pH 7.4). For analytical SEC, 100 μl of antibodies (diluted to 0.1 mg/ml) were loaded onto an SEC column (Superdex 200 Increase 10/300 GL column; GE, 28990944) and analyzed at 0.75 ml/min using a PBS running buffer supplemented with 200 mM arginine (pH 7.4). Absorbance was monitored at 220 and 280 nm, and the 280 nm signal was primarily used for analysis. The percentage of antibody monomer was evaluated by analyzing the area under the monomeric peak (excluding times before 7 min and after 22 min). In some cases, the antibodies were purified using SEC after Protein A purification. In those cases, the peak times for fraction collection were chosen based on the analytical runs. Antibody fractions were collected, buffer exchanged into PBS (pH 7.4), filtered, aliquoted, and stored at −80 °C.

### Antibody binding analysis

For affinity analysis, the binding of antibodies [including clinical-stage antibodies whose sequences were obtained from the Therapeutic Antibody (TABS) database] to Aβ fibrils was evaluated using streptavidin dynabeads and flow cytometry. Beads were immobilized with 1% biotinylated fibrils as described above. The fibril-coated beads were washed twice with PBSB and then blocked with 10% milk in PBS at room temperature for 1 h with end-over-end mixing. Afterward, the beads were washed 2x with PBSB.

Antibodies were thawed and centrifuged at 21,000*g* for 5 min to remove aggregates. The supernatant was transferred to a new tube and the antibody concentration was determined by measuring absorbance at 280 nm. Antibody dilutions were made in PBSB. Fibril-coated beads (1.25 × 10^5^ beads per antibody concentration) were incubated with antibodies in 96-well plates (Greiner, 650261) in 1% milk for 3 h at 25 °C (300 RPM). Next, the plates were centrifuged at 3500 RPM for 5 min, the supernatants were discarded, and the beads were washed once with ice-cold PBSB. After washing, the plates were spun down again and the beads were resuspended with 300x diluted goat anti-human Fc AF647 (Jackson Immunoresearch, 109-605-098) on ice for 4 to 5 min. Beads were then washed once more with ice-cold PBSB and analyzed *via* flow cytometry using a BioRad ZE5 Analyzer. For control beads, blank streptavidin beads were also blocked with 10% milk in PBS and treated in the same way as the fibril-coated beads. Two independent repeats were performed with different batches of beads coated with Aβ fibrils.

For antibody conformational specificity analysis, the experiments were performed in the same way as described above except that the antibodies were preincubated with disaggregated (nonbiotinylated) Aβ. Antibody binding analysis was performed in 1% milk at a fixed antibody concentration (30 nM) and a range of disaggregated Aβ concentrations. The antibody binding results were normalized to the average value obtained without disaggregated Aβ. Two independent repeats were performed with different batches of beads coated with Aβ fibrils.

### Antibody epitope analysis

Fibrils were also assembled using Aβ peptides with N-terminal deletions including Aβ2-42 (Bachem, 40306028.0500), Aβ3-42 (Bachem, 4090137.0500), Aβ4-42 (Bachem, 4090138.0500), Aβ5-42 (Bachem, 4041241.0500), and Aβ11-42 (Anaspec, 63317) in addition to Aβ1-42 and purified using ultracentrifugation. Fibrils were then spotted on nitrocellulose membranes at equal Thioflavin T florescence. Membranes were blocked with 5% milk in PBS at room temperature for 1 h followed by 3x washing with PBST (PBS with 0.1% Tween 20). Membranes were then incubated with Aβ antibodies at 10 nM (1% milk) in PBST at room temperature for 2 to 3 h. Following primary incubation, membranes were washed 3x with PBST followed by incubation with goat anti-human Fc IgG HRP (1/5000x dilution, Invitrogen, A18817) in PBST at room temperature (1 h). Following secondary incubation, the blots were washed 3x with PBST, developed with ECL (Pierce, 32109), and imaged with a BioRad imager.

### Polyspecificity analysis

The polyspecificity reagent (PSR) was prepared as previously described (51). CHO cells (10^9^, Gibco, A29133) were pelleted, the cell pellets were washed separately with PBSB and Buffer B (50 mM HEPES, 0.15 M NaCl, 2 mM CaCl_2_, 5 mM KCl, 5 mM MgCl_2_, 10% Glycerol, pH 7.2) and then pelleted again. The pellets were resuspended in 5 ml of Buffer B supplemented with a protease inhibitor (Sigma Aldrich, 4693159001). Next, the resuspended cells were homogenized for 90 s (three cycles of 30 s) followed by sonication for 90 s (three cycles of 30 s). The cell suspension was then spun down at 40,000*g* for 1 h and the supernatant was discarded.

The pellet, comprising the enriched membrane fraction, was resuspended in Buffer B with a Dounce homogenizer for 30 strokes. The protein concentration was determined using a detergent compatible protein assay kit (BioRad, 5000116). The enriched membrane fraction was diluted to a theoretical concentration of 1 mg/ml in solubilization buffer (pH 7.2), the latter of which contained 50 mM HEPES, 0.15 M NaCl, 2 mM CaCl_2_, 5 mM KCl, 5 mM MgCl_2_, 1% n-dodecyl-β-D-maltopyranoside (Sigma Aldrich, D4641), and a protease inhibitor (Sigma Aldrich, 11873580001). The solution was then mixed overnight (end-over-end) at 4 °C. The soluble membrane protein fraction was centrifuged at 40,000*g* for 1 h and the supernatant was collected. The final concentration of supernatant was ∼0.8 to 0.9 mg/ml.

Sulfo-NHS-LC-biotin (Thermo Fisher, PI21335) was dissolved in distilled water at ∼11.5 mg/ml. The stock solution of Sulfo-NHS-LC-biotin (150 μl) and the PSR reagent (4.5 ml at 0.8–0.9 mg/ml) were mixed *via* end-over-end mixing at room temperature (45 min). The reaction was quenched (10 μl of 1.5 M hydroxylamine at pH 7.2), and biotinylated PSR was aliquoted and stored at −80 °C.

Protein A magnetic beads (Invitrogen, 88846) were washed twice and incubated with antibodies in 96-well plates (VWR, 650261) overnight at 4 °C. The antibodies were purified either *via* one-step (Protein A) or two-step (Protein A followed by SEC) purification methods. Next, the antibody-coated beads were washed by centrifuging the 96-well plates at 3500*g* for 4 min and washed twice with PBSB. Afterward, the beads were resuspended with a 10x diluted solution of biotinylated PSR and incubated on ice for 20 min. Beads were washed once with PBSB and incubated with 1000x diluted solution of streptavidin AF-647 (Invitrogen, S32357) and 1000x diluted solution of goat anti-human Fc F(ab’)_2_ AF-488 (Invitrogen, H10120) on ice (4 min). Bead were washed once, resuspended in PBSB, and analyzed *via* flow cytometry. The antibody binding steps were performed in PBSB, and three independent repeats were performed. The control antibodies used in these experiments possessed the variable regions of crenezumab, elotuzumab, duligotuzumab, and emibetuzumab grafted onto a common IgG1 framework, which results in differences in the antibodies we have evaluated and the actual clinical-stage drugs. The control antibodies were two-step purified (Protein A and SEC).

### Immunoblotting analysis of synthetic Aβ peptides

For immunoblots using synthetic Aβ peptides, disaggregated Aβ and unlabeled Aβ fibrils were prepared as discussed above. Disaggregated Aβ and fibrils of Aβ, IAPP, and α-synuclein were spotted on nitrocellulose membranes. Membranes were allowed to dry for at least 1 h at room temperature before use. Membranes were blocked with 5% milk in PBS for 1 h at room temperature. Afterward, the membranes were washed 3x using PBST (PBS with 0.1% v/v Tween 20) with rocking (5 min). Antibodies were thawed, centrifuged, and their concentrations were determined *via* absorbance measurements at 280 nm. Antibody binding was performed at 10 nM in PBST with 1% milk at room temperature (3 h). Next, the membranes were washed 3x with PBST and incubated with a 7500x diluted solution of goat anti-human Fc HRP (Invitrogen, A18817) at room temperature (1 h). Following secondary incubation, the blots were washed 3x with PBST and developed with ECL (Pierce, 32109). The signals were evaluated using X-Ray film (Thermo Scientific, 34090) and the films were developed. Three independent repeats were performed for all experiments.

### Mouse models

This study was conducted in a facility approved by the American Association for the Accreditation of Laboratory Animal Care, and all experiments were performed in accordance with the National Institutes of Health Guide for the Care and Use of Laboratory Animals and approved by the Institutional Animal Care and Use Committee of the University of Michigan. Mice were housed at the University of Michigan animal care facility and maintained according to U.S. Department of Agriculture standards (12 h light/dark cycle with food and water available *ad libitum*). 5xFAD mice (B6.Cg_Tg(APPSwFlLon,PSEN1∗M146L∗L286V)6799Vas/Mmjax; The Jackson Laboratory MMRRC stock #034848) expressing human amyloid precursor protein (APP) and presenilin-1 (PSEN1) with five AD mutations: the Swedish (K670N/M671L), Florida (I716V), and London (V717I) APP mutations, and the M146L and L286V PSEN1 mutations and nontransgenic littermates (courtesy of Geoffrey Murphy, University of Michigan) were euthanized at 8 months (for immunofluorescence analysis) and 22 to 24 months (for immunoblots and western blots) for brain collection.

### Tissue harvesting

Animals were deeply anesthetized with isofluorane and perfused transcardially with 1x PBS. Brains were divided sagittally. One half was immediately placed on dry ice and stored at −80 °C for biochemical studies while the other half was fixed in 4% paraformaldehyde at 4 °C for 24 h and cryoprotected in 10% and 30% sucrose solutions in 1xPBS at 4 °C until saturated. Fixed hemispheres were snap frozen in OCT medium and sectioned at 12 μm sagittally using a cryostat and sections were stored at −20 °C for immunofluorescence.

### Immunoblotting and western blotting analysis of mouse brain samples

The 5xFAD and nontransgenic littermate forebrain samples were homogenized in PBS with a protease inhibitor cocktail (Sigma Aldrich, 11873580001) using a 1:3 dilution of tissue: PBS (w/v). Samples were centrifuged at 9300*g* for 10 min at 4 °C. Supernatants (soluble fraction) were snap frozen and stored at −80 °C for western blot analysis. Pellets were resuspended in PBS with protease inhibitor cocktail (Roche, 11836170001), centrifuged at 9300*g* for 10 min (4 °C), and supernatants were discarded. The pellet was resuspended in radioimmunoprecipitation assay (RIPA) buffer with protease inhibitor, vortexed (1 min), and incubated at room temperature (1 h). Samples were sonicated (water bath sonicator) for 5 min and centrifuged for 30 min (16,000*g* at 4 °C). RIPA (PBS soluble and insoluble) fractions of brain extracts (7 μg of total protein) were spotted directly onto nitrocellulose membranes and allowed to dry (1 h). Control dot blots (loading controls) were stained with Ponceau S (5 min) and washed 3x with distilled water. The other dot blots were blocked with 10% nonfat dry milk in Tris Buffered Saline with 0.1% Tween 20 (TBST) buffer at room temperature (1 h). Each dot blot was then incubated with antibodies at 50 nM (1% nonfat dry milk in TBST) overnight at 4 °C. Next, the blots were washed with TBST and incubated with a 5000x diluted solution of HRP-conjugated goat anti-human IgG at room temperature for 1 h. Afterward, the blots were washed with TBST and developed using Ecobright Nano HRP Substrate (Innovative Solutions) and visualized with the Genesys G:Box imaging system (Syngene). Three independent repeats were performed.

For western blotting, 50 μg of total protein was loaded on precast NuPAGE 4 to 12% Bis-Tris gels (Invitrogen, WG1402A). Gels were subsequently transferred onto nitrocellulose membranes and first stained with Ponceau S and washed 3x with distilled water. After imaging, membranes were destained for 1 min with 0.1 M NaOH and washed 3x with distilled water. Next, membranes were blocked for 1 h at room temperature with 10% nonfat dry milk in TBST buffer. Membranes were probed overnight at 4 °C with aducanumab (Adu) and NAB 228 (Sigma-Aldrich, A8354; recognizes Aβ1-11) at 100 nM in TBST with 1% milk or 100 nM antibody (clone 93 or 97) in 1% nonfat dry milk in TBST. HRP-conjugated goat anti-human/mouse IgG (5000x dilution) HRP was used for detection. Ecobright Nano HRP Substrate (Innovative Solutions) was used to visualize bands with the Genesys G:Box imaging system (Syngene). Three independent repeats were performed.

### Human disease brain tissue

Frozen brain tissue from the hippocampus of subjects with Alzheimer's disease and age-matched control subjects from the Michigan Brain Bank (University of Michigan, Ann Arbor, MI, USA). Brain tissue was collected with the informed consent of the patients. Protocols were approved by the Institutional Review Board of the University of Michigan and abide by the Declaration of Helsinki principles. Samples were examined at autopsy by neuropathologists for diagnosis.

### Processing of human brain tissues

Lysis buffer (600 μl; 50 mM Na phosphate, pH 7.4, 150 mM NaCl, 2 mM EGTA, 2 mM EDTA, PhosSTOP (Sigma-Aldrich; 4906845001), cOmplete EDTA-free Protease Inhibitor Cocktail mini (Sigma-Aldrich; 11836170001), 6 μl/ml saturated phenylmethylsulfonyl fluoride (PMSF), and 10 mM Na azide) were added to 0.3 g of hippocampal tissue from individuals diagnosed with AD and age-matched controls negative for Aβ, α-synuclein, and tau pathology. Next, tissue samples were homogenized in safe-lock tubes containing four zirconium beads per tube for 1 min (speed 4) followed by cooling on ice for 5 min (Nova Advance homogenizer, Next Advance). Homogenization was repeated three more times. For additional homogenization, samples were passaged five times through a 25G needle, followed by centrifugation at 1,000*g* for 10 min. After resuspension with 150 μl lysis buffer, pellets were passaged five times through a 25G needle syringe. Next, samples were sonicated (PIP 50, DF 10%, and CPB 200) for 100 cycles (1 s ON and 1 s OFF) in M220 Focused-ultrasonicator (Covaris). To digest RNA/DNA, 1 μl benzonase (Sigma; E1014) was added to 50 μl sample (1:50 ratio) supplemented with 2 mM MgCl_2_ (final concentration). After incubation for 30 min at room temperature, equal volumes of benzonase-treated samples and 1% sarkosyl were incubated for 30 min at 4 °C. Following centrifugation at 18,000*g*, the total protein concentrations of the pellets were determined by BCA and used for the dot blots.

### Immunofluorescence analysis of mouse brain samples

Fixed brain sections were postfixed for 10 min in methanol at 4 °C. Sections were washed three times in 1x PBS for 10 min and subjected to heat-induced antigen retrieval in 10 mM citrate buffer (pH 6). Sections were washed two times in 1x PBS for 5 min and permeabilized with 0.5% Triton-X 100, washed for 10 min in 1xPBS, and blocked using the Mouse on Mouse (M.O.M.) Mouse IgG Blocking Reagent (M.O.M. Immunodetection Kit, Vector, BMK-2202) for 1 h. Sections were washed 2x for 2 min in 1x PBS and incubated for 5 min in M.O.M. diluent. Sections were then incubated with Aβ antibodies aducanumab or 97 (200 nM) and NAB 228 (200x dilution) in M.O.M. diluent overnight at 4 °C. The following day, sections were washed in 1x PBS three times for 10 min each and incubated with goat anti-mouse IgG Alexa-488 (Invitrogen; 1:500) and goat anti-human IgG Alexa-647 (1:500) for 1 h. Sections were then washed in PBS 3x for 10 min each and incubated with DAPI (Sigma) to label nuclei for 5 min at room temperature, washed 3x for 5 min each, and mounted with Prolong Gold Antifade Reagent (Invitrogen). Slides were imaged using a Leica SP5 Confocal microscope.

## Data availability

All of the data is contained within the article except for the antibody sequences, which are deposited in GenBank: MT635022, MT635023, MT635024, MT635025, MT635026, MT635027, MT635028, MT635029, MT635030, MT635031, MW202274.

## Supporting information

This article contains supporting information.

## Conflict of interest

The authors declare that they have no conflicts of interest with the contents of this article.
