# Supplementary material for: Rational affinity maturation of anti-amyloid antibodies with high conformational and sequence specificity
Source: J Biol Chem. 2021 Mar 4;296:100508. doi: 10.1016/j.jbc.2021.100508 (PMC8081927; doi:10.1016/j.jbc.2021.100508)
Supplement: Supplemental Figures S1–S11 and Table S1 [file mmc1.pdf]

Table S1. **Theoretical net charges and isoelectric points for clinical-stage antibodies relative to the antibodies in this work.** The net charges were calculated at pH 7.4. H123 represents the three heavy chain CDRs, while L123 represents the three light chain CDRs. The charges were calculated for CDRs using Kabat numbering except for H1 (combined Chothia and Kabat defintion) and H3 (Kabat defintion plus two additional N-terminal residues after cysteine).

		**Theoretical charge at pH 7.4**												**Theoretical pI**		
**Antibody**	**Antigen**	**VH**	**VL**	**Fv**	**CDR**	**H1**	**H2**	**H3**	**H123**	**L1**	**L2**	**L3**	**L123**	**VH**	**VL**	**Fv**
Aducanumab	Aβ	7.1	2.0	9.1	4.1	0.1	1	2	3.1	1	0	0	1	9.7	8.6	9.4
Gantenerumab	Aβ	5.1	1.0	6.1	6.1	0	1	3.1	4.1	1	1	0	2	9.4	8.0	9.1
Emibetuzumab	HGFR	4.1	1.1	5.2	2.2	-0.9	3	0	2.1	0.1	0	0	0.1	9.2	8.0	9.0
Ponezumab	Aβ	2.1	2.1	4.2	1.2	-0.9	1	0	0.1	1	0	0.1	1.1	8.6	8.7	8.8
Bapineuzumab	Aβ	2.1	0.1	2.2	2.2	0	2	-0.9	1.1	0	0	1.1	1.1	8.6	6.8	8.3
BAN2401	Aβ	1.1	1.2	2.3	2.3	0.1	0	0	0.1	0.1	2	0.1	2.2	8.0	8.7	8.6
Solanezumab	Aβ	1	2.2	3.2	2.2	1	0	-1	0	0.1	2	0.1	2.2	8.1	8.7	8.7
Crenezumab	Aβ	0	0.2	0.2	1.2	0	0	-1	-1	0.1	2	0.1	2.2	6.4	6.9	7.0
Duligotuzumab	HER3	-3.9	-1.0	-4.9	-5.9	-0.9	-2	-1	-3.9	0	0	-2	-2	4.8	5.2	4.9
Elotuzumab	CD319	-2	1.1	-0.9	-1.9	0	-2	-1	-3	0	1.1	0	1.1	5.0	8.0	5.9
AF1	Aβ	-1.9	2.1	0.2	-1.9	0.1	2	-4	-1.9	0	0	0	0	5.2	8.6	7.0
88	Aβ	-0.9	3.1	2.2	0.1	0.1	3	-4	-0.9	1	0	0	1	5.7	9.0	8.3
89	Aβ	-0.9	3.1	2.2	0.1	0.1	3	-4	-0.9	1	0	0	1	5.7	9.0	8.3
91	Aβ	-0.9	3.1	2.2	0.1	0.1	3	-4	-0.9	1	0	0	1	5.7	9.0	8.3
92	Aβ	-0.9	3.1	2.2	0.1	0.1	3	-4	-0.9	1	0	0	1	5.7	9.0	8.3
93	Aβ	-0.9	3.1	2.2	0.1	0.1	3	-4	-0.9	1	0	0	1	5.7	9.0	8.3
95	Aβ	-0.9	3.1	2.2	0.1	0.1	3	-4	-0.9	1	0	0	1	5.7	9.0	8.3
97	Aβ	-1.9	3.1	1.2	-0.9	0.1	2	-4	-1.9	1	0	0	1	5.2	9.0	7.8
98	Aβ	-0.9	3.1	2.2	0.1	0.1	3	-4	-0.9	1	0	0	1	5.7	9.0	8.3
101	Aβ	-0.9	3.1	2.2	0.1	0.1	3	-4	-0.9	1	0	0	1	5.7	9.0	8.3
102	Aβ	-0.9	3.1	2.2	0.1	0.1	3	-4	-0.9	1	0	0	1	5.7	9.0	8.3
97A3	Aβ	-1.9	4.1	2.2	0.2	0.1	2	-4	-1.9	1	1	0.1	2.1	5.2	9.2	8.3

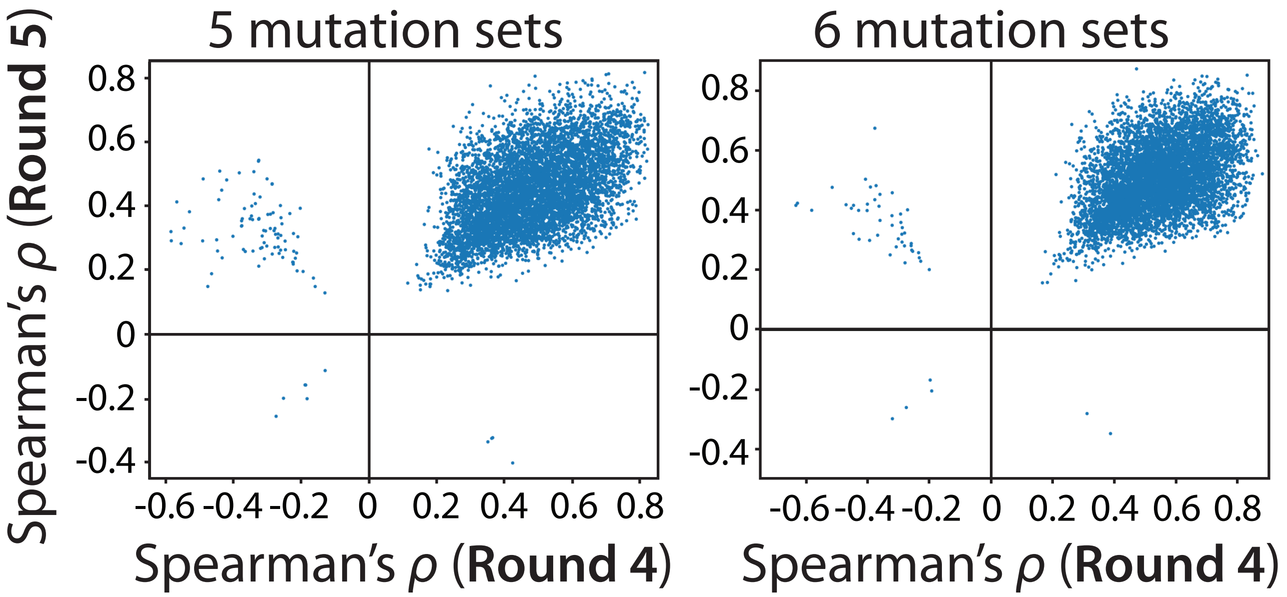

Figure S1. Comparison of Spearman correlation coefficients for sets of mutations identified in multiple rounds of antibody library sorting against Aβ fibrils. Antibody libraries were sequenced and sets of mutations were identified as described in Figure 4. All possible combinations of five and six mutation sets were evaluated, and only those that were statistically significant (p-value <0.05) in both rounds 4 and 5 are reported. The percentages of sets of mutations with Spearman correlation coefficients that were either both positive or both negative in rounds 4 and 5 were 98.6% (5 mutation sets) and 99.3% (6 mutation sets).

	HCDR2					LCDR1				LCDR3		Spearman's correlation			
**Antibody**	52	53	54	56	61	28	30	31	34	92	94	Round 4		Round 5	
**WT**	**Y**	**T**	**N**	**Y**	**D**	**D**	**N**	**T**	**A**	**Y**	**T**	ρ	*p* - value	ρ	*p* - value
99		A		N			A	Y			Y	0.81	3E-08	0.82	4E-10
97				A		N	A	Y			Y	0.80	9E-15	0.67	1E-09
100				N	A		A	Y			Y	0.76	2E-08	0.78	6E-09
95		A				N		S	T		S	0.77	2E-10	0.71	2E-08
102					A	N	A	S			Y	0.69	1E-09	0.73	7E-12
89		Y		A		N		S			Y	0.79	7E-07	0.65	2E-05
98		S			A		A	Y			Y	0.71	1E-06	0.78	1E-08
96		Y				N	A	A			Y	0.80	2E-11	0.69	3E-08
92					A	N	Y	S			Y	0.65	1E-07	0.78	3E-12
93					G	N		N	T		Y	0.76	2E-12	0.67	5E-10
101		A			G	N		S			Y	0.70	1E-06	0.71	2E-06
91					G	N		S			Y	0.70	1E-06	0.71	2E-06
88		N			G	N	Y		T		Y	0.63	9E-07	0.63	6E-07
94			S			N	A		S		S	0.69	3E-15	0.52	6E-09
90		A				N	Y	S			Y	0.75	1E-08	0.62	3E-05

Figure S2. Antibody variants with sets of five mutations that display strong correlation with improved enrichment for recognizing Aβ fibrils relative to wild-type (AF1). The mutation sets were identified as described in Figure 4. There are three to five additional mutations not shown for each antibody variant because they are not one of the five mutations most correlated with improved enrichment ratios. The color codes for the amino acids are described in Fig. 2.

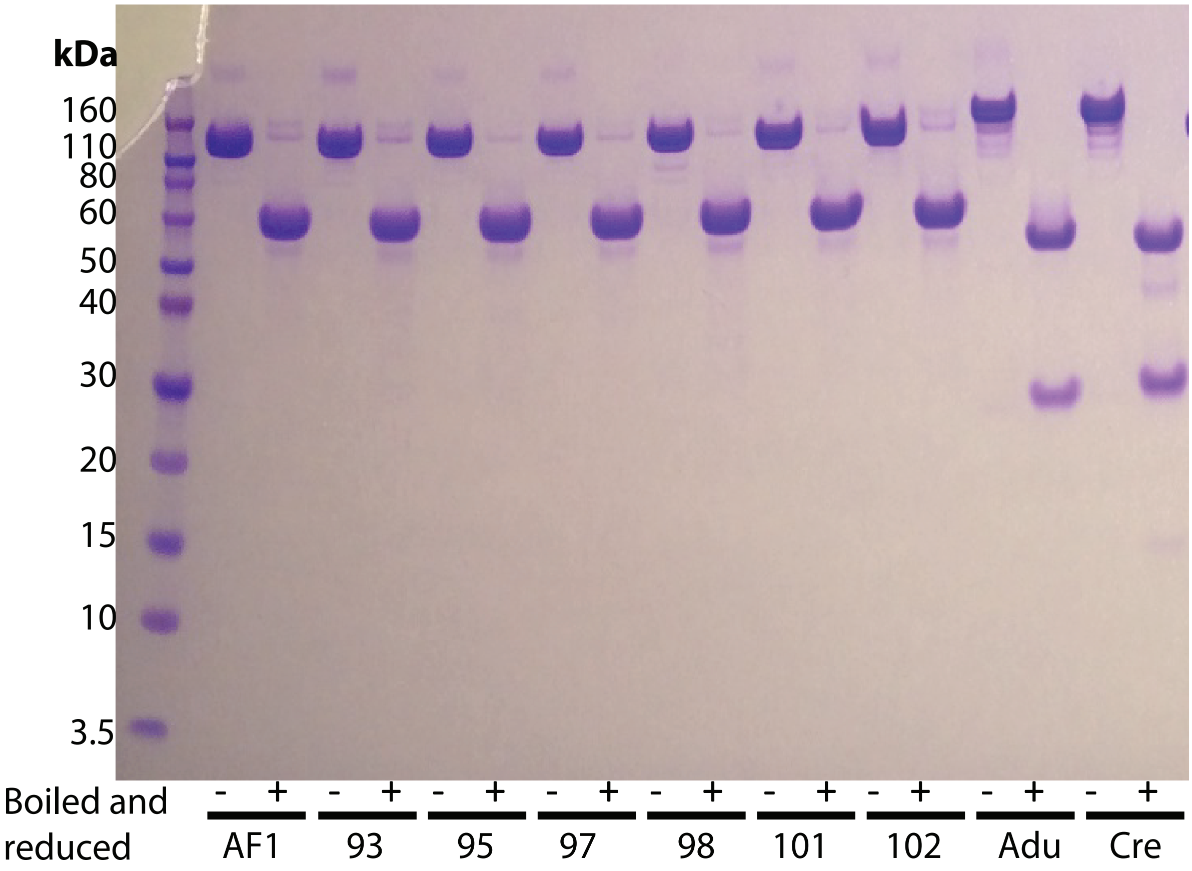

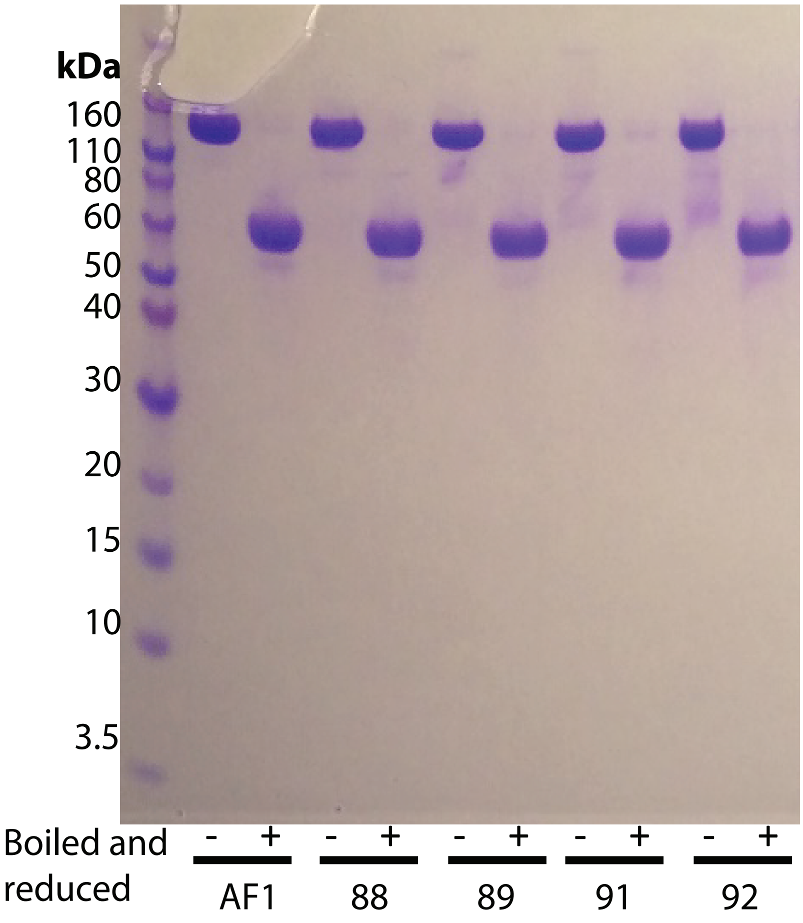

Figure S3. SDS-PAGE analysis of the Aβ antibodies evaluated in this study. Antibodies were evaluated prior to boiling and reduction (-) and after boiling and reduction (+). The gels (10% Bis-Tris) were visualized using Coomassie blue staining.

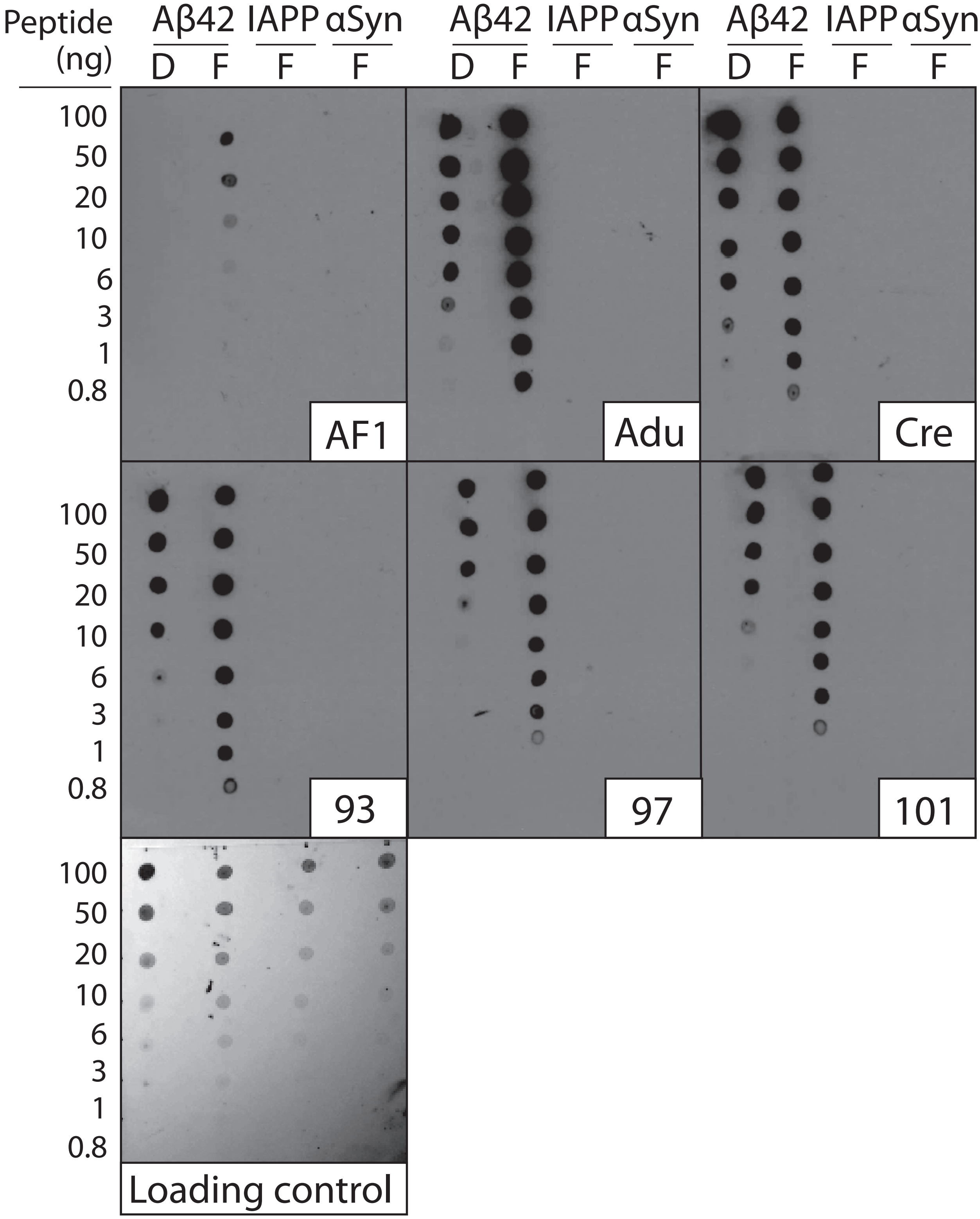

Figure S4. Immunoblot analysis of the conformational and sequence specificity of the selected Aβ antibodies for an extended X-ray film exposure. Blots from Fig. 6 were processed and also imaged after a relatively long exposure time (45 min). The Ponceau S stained loading control is the same as in Fig. 6 because the same blots stained with antibodies are shown after a longer exposure time. The experiments were repeated three times and a representative example is shown.

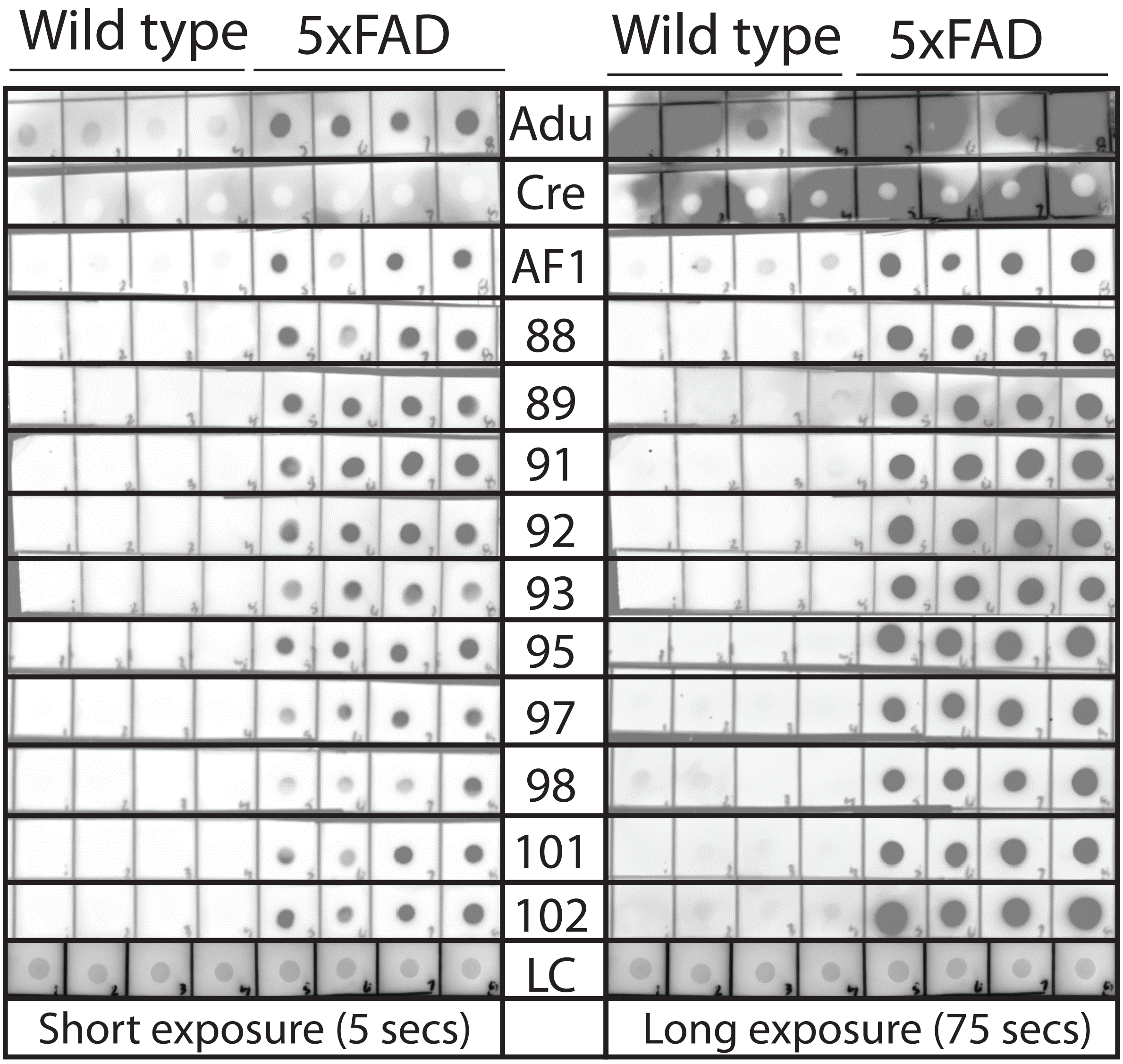

Figure S5. Immunoblot analysis of transgenic (5xFAD) and wild-type mouse brain samples using Aβ antibodies. Brain samples (insoluble fraction) obtained from 5xFAD (22-24 months old) and wild-type mice were immobilized on nitrocellulose membranes and probed with Aβ antibodies (50 nM in TBST with 1% milk), including aducanumab (Adu) and crenezumab (Cre). The blots were imaged after relatively short (~5 s) and long (~75 s) exposures. Ponceau S staining was used as a loading control (LC). The loading control is the same as in Fig. 8 because the same batch of blots (prepared at the same time) that were stained with antibodies were processed with different exposure times. The experiments were repeated three times and a representative example is shown.

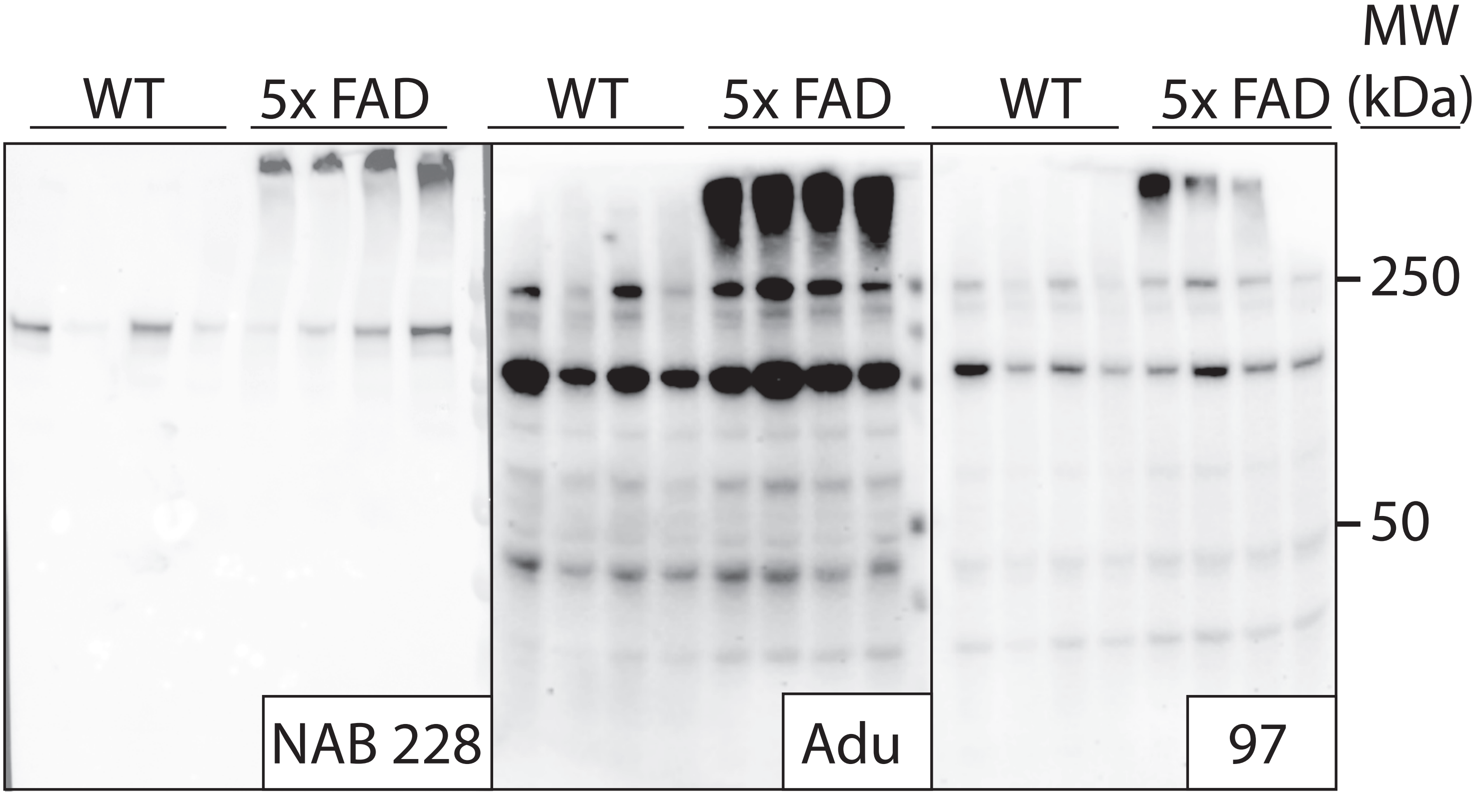

Figure S6. Western blot analysis of transgenic (5xFAD) and wild-type mouse brain samples using Aβ antibodies. Brain samples (PBS soluble fraction) isolated from 5xFAD (22-24 months old) and wild-type (WT) mice were processed via SDS-PAGE (boiled samples), transferred to nitrocellulose membranes, and probed with a subset of Aβ antibodies (50 nM in TBST with 1% milk). The blots were imaged after ~13 min of exposure. The experiments were repeated three times and a representative example is shown.

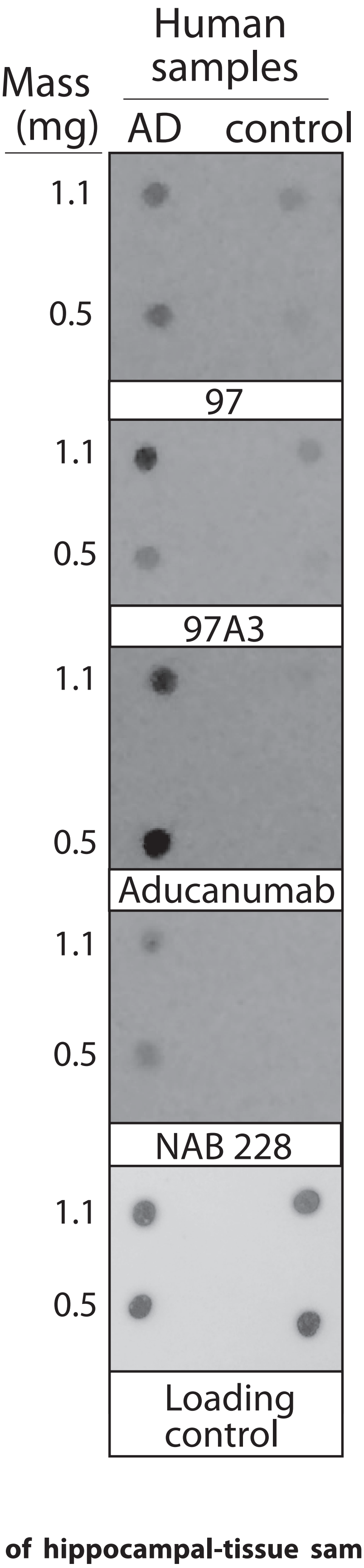

Figure S7. Immunoblot analysis of hippocampal-tissue samples of individuals with Alzheimer's disease and age-match controls using Aβ antibodies. Brain-tissue samples (sarkosyl insoluble fraction of lysed hippocampus) obtained from patients with Alzheimer's disease (AD) and healthy individuals (controls, absence of pathology positive for Aβ, α-synuclein or tau) were immobilized on nitrocellulose membranes and probed with Aβ antibodies (97, 97A3 at 10 nM in PBST with 1% milk), including aducanumab (Adu) [1 nM in PBST with 1% milk] and a sequence-specific Aβ antibody (NAB 228). The control sample is detected using colloidal silver stain. These experiments were repeated 2-3 times and a representative example is shown.

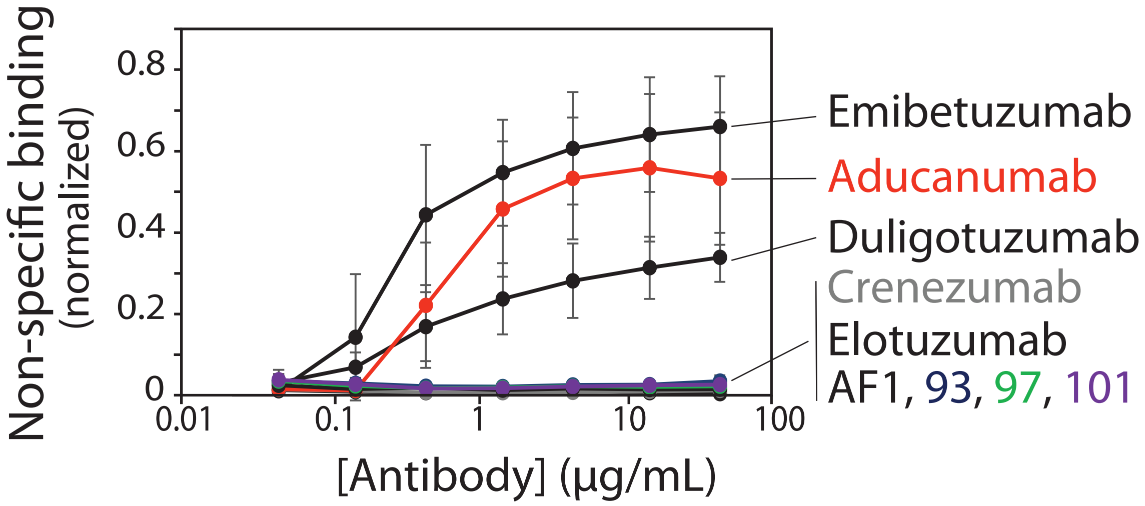

Figure S8. Non-specific binding analysis of Aβ antibodies purified using both Protein A and size-exclusion chromatography. The non-specific binding results were performed as described in Figure 11 except that the antibodies were purified using both Protein A and size-exclusion chromatography. The values are averages and the error bars are standard deviations (three independent repeats).

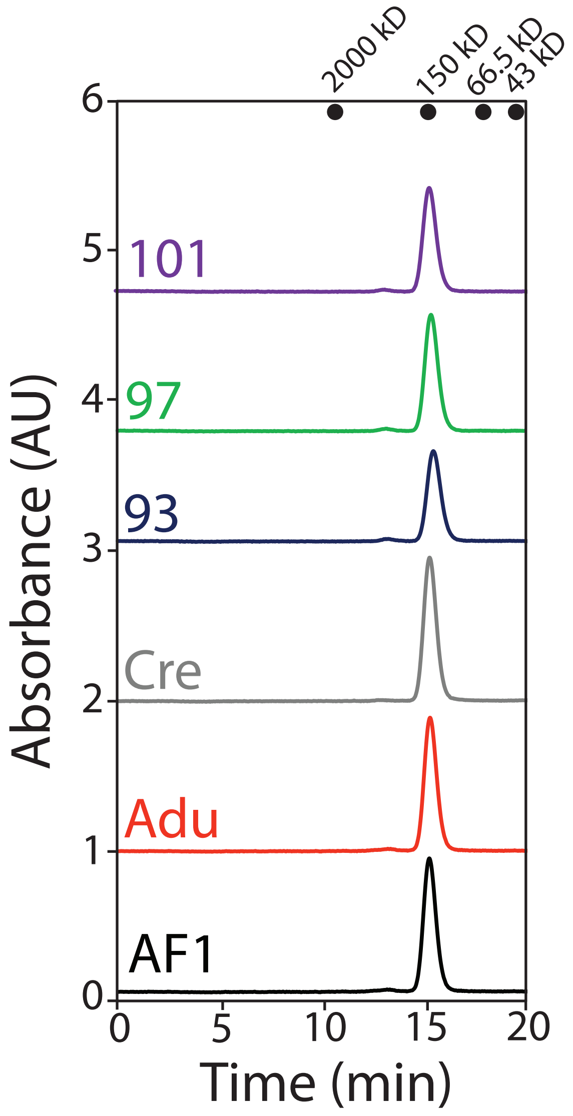

Figure S9. Analytical size-exclusion chromatography analysis of Aβ antibodies. Representative chromatograms for Aβ antibodies after Protein A purification. The running buffer was PBS with 200 mM arginine (pH 7.4).

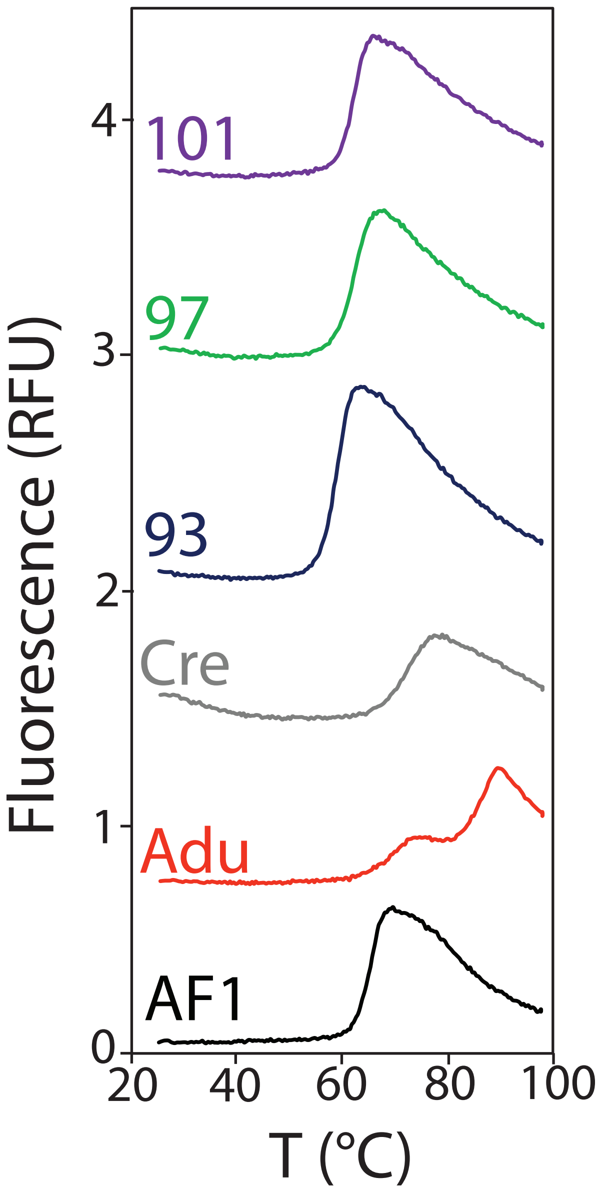

Figure S10. Antibody thermal unfolding curves evaluated using differential scanning fluorimetry. Representative antibody unfolding curves monitored using the Thermal Shift dye.

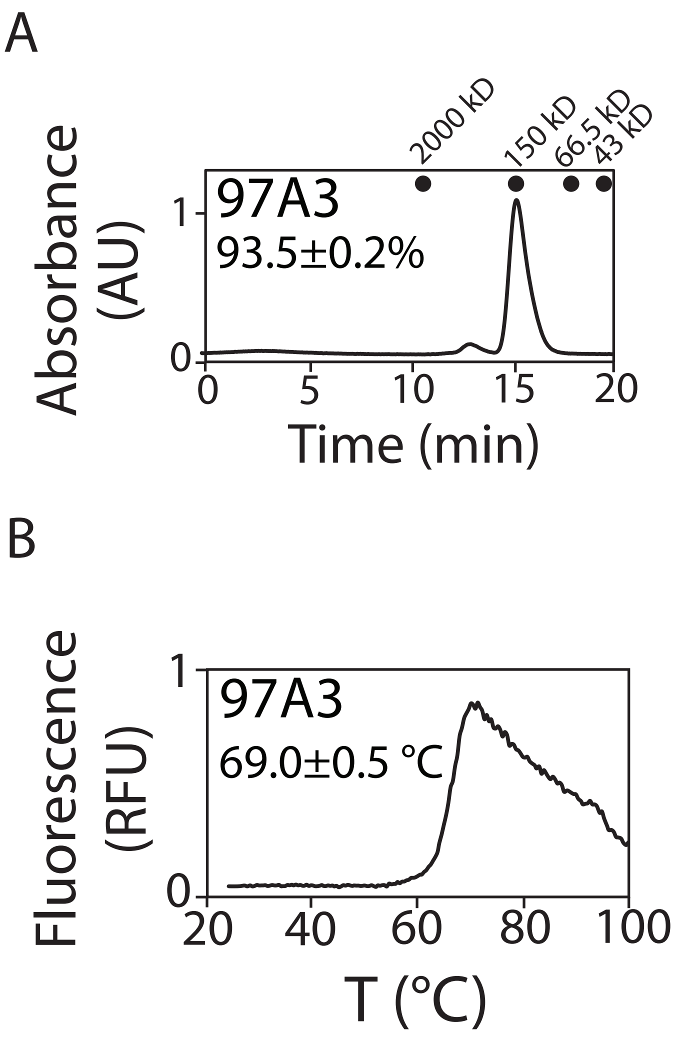

Figure S11. Analytical size-exclusion chromatography analysis and thermal unfolding curves for an affinity-matured Aβ antibody (97A3). (A) Representative chromatograms for Aβ antibody 97A3 after Protein A purification. The running buffer was PBS with 200 mM arginine (pH 7.4). (B) Representative thermal unfolding curve for clone 97A3 using a fluorescent thermal shift dye. The experiments were repeated three times and a representative example is shown.
